# *TWIST1* mediated transcriptional activation of *SPON2* drives colorectal cancer peritoneal metastasis through stromal cell signaling network

**DOI:** 10.1038/s41388-026-03743-7

**Published:** 2026-04-03

**Authors:** Zhuan Zhou, Alessandro La Ferlita, Manoj H. Palavalli, Xiongfeng Chen, Lauren Tyler, Aslam Ejaz, Joal Beane, Patricio M. Polanco, Huocong Huang, Alex C. Kim

**Affiliations:** 1 Division of Surgical Oncology, Department of Surgery, UT Southwestern Medical Center, Dallas, TX USA; 2 Division of Medical Oncology, Department of Medicine, The Ohio State University Wexner Medical Center, the James Comprehensive Cancer Center and Solove Research Institute, Ohio State University, Columbus, OH USA; 3 Division of Surgical Oncology, Department of Surgery, The Ohio State University Wexner Medical Center, the James Comprehensive Cancer Center and Solove Research Institute, Ohio State University, Columbus, OH USA; 4 Department of Surgery, Section of General Surgery and Surgical Oncology, University of Illinois-Chicago, Chicago, IL USA

**Keywords:** Metastasis, Cancer microenvironment, Colorectal cancer

## Abstract

Colorectal cancer (CRC) peritoneal metastasis (PM) accounts for 25–35% of stage IV cases. CRC PM carries a median overall survival of 16 months with systemic chemotherapy and an almost 0% 5-year survival rate. The molecular mechanisms driving CRC PM remain poorly defined. CRC heterogeneity is classified into four Consensus Molecular Subtypes (CMS1-4), with CRC PM predominantly exhibiting the CMS4 signature—characterized by increased stromal/mesenchymal enrichment and cellular plasticity—features linked to frequent disease progression and therapeutic resistance. Here, we investigated the molecular mechanisms driving CRC PM and CMS4 signature. TWIST1 was identified to be significantly upregulated in CRC PM. We established TWIST1-SPON2 as a novel transcriptional axis contributing to CRC PM tumorigenesis, through mediating tumor-stroma interactions. We identified SPP1, secreted by the tumor stroma, as an upstream regulator of the TWIST1-SPON2 cascade via AKT activation in tumor cells in vitro and in vivo. This defined SPP1-TWIST1-SPON2 signaling circuit is pivotal in shaping the tumor microenvironment and promoting CRC PM progression. The findings establish the SPP1-TWIST1-SPON2 axis as potential biomarkers and therapeutic targets in CRC PM.

## Introduction

Colorectal cancer (CRC) is the third most common cancer diagnosis and second leading cause of cancer death in the United States [1]. Among its metastatic patterns, peritoneal metastasis (PM) represents a particularly aggressive and challenging form of disease dissemination, affecting approximately 20–30% of CRC patients and comprising 25–35% of stage IV cases [2–4]. PM is associated with the poorest prognosis among metastatic CRC sites, with a median survival of only 6 months without systemic therapy and 16 months with systemic therapy [2, 5]. Post-mortem studies show PM in nearly 50% of CRC patients, suggesting it is a large driver of patient mortality [6]. Current curative-intent strategies for selected patients involve cytoreductive surgery (CRS) combined with hyperthermic intraperitoneal chemotherapy (HIPEC). While this multimodal approach has improved survival for specific subgroups, it is associated with significant perioperative morbidity. Furthermore, its efficacy is often limited by the completeness of cytoreduction, and the majority of patients eventually experience recurrence (50–90%) due to microscopic residual disease and resistance to standard chemotherapeutics, contributing to a dismal 5-year overall survival rate approaching 0% [7, 8]. With the rising incidence of advanced CRC, particularly early-onset cases (<50 years old), PM represents a growing healthcare burden [1]. Notably, the molecular mechanisms underlying CRC PM remain poorly understood.

A historical paradigm for developing CRC PM envisaged a breach of the colonic wall by T4 (obstructed or perforated) tumors, subsequent exfoliation, and direct peritoneal seeding. However, only 16–19% of T4 patients experience PM, an observation that appears more consistent with the conclusion that PM also arises from T1-3 tumors [9–11]. These results suggest that a distinct unknown molecular program may drive PM throughout the progression of CRC.

The Consensus Molecular Subtype (CMS) classification system categorizes CRC into distinct molecular subgroups, with the CMS4 subtype exhibiting the worst prognosis due to frequent therapeutic resistance and poor relapse-free survival [12]. PMs are highly enriched for the CMS4 signature, while colorectal cancer liver metastasis (CRLM) are represented by heterogeneity of CMS1-4 signatures [13]. CMS4 attributes, characterized by upregulation of genes promoting epithelial-to-mesenchymal transition (EMT), stromal enrichment, and stemness, are strongly associated with high tumor burden and resistance to standard therapies [13].

EMT, a key developmental program aberrantly activated in many cancers including CMS4 CRC and PM, enhances tumor cell plasticity and metastatic potential. EMT is regulated by well-established transcription factors such as TWIST1, SNAI1, SNAI2, and ZEB1. Among these, TWIST1 is a known high-risk factor for CRC metastasis that includes lymph node involvement and tumor budding [14, 15]. Earlier studies in CRC demonstrated that the stroma of CRC fostered TWIST1-postive cancer cells with robust mesenchymal phenotype at the invasion front [16]. Interestingly, TWIST1 activation was specifically observed in undifferentiated CD44^+^ primary CRC cells, following paracrine stimulation arising from the stroma [17]. In this study, we demonstrate the upregulation of TWIST1 in CRC PM and that TWIST1 knockout significantly reduces metastatic potential by impairing migration, invasion, and spheroid formation.

Through integrative bioinformatic analyses utilizing chromatin immunoprecipitation sequencing (ChIP-Seq) and RNA sequencing (RNA-Seq), we identified SPON2 as a direct downstream TWIST1 target crucial for CRC PM tumorigenesis and tumor microenvironment remodeling. Peritoneal mesothelial cells are hypothesized to be the first cell layer encountered tumor cells during PM. Furthermore, mesothelial cells are thought to promote PM progression [18]. Consistent with this, we recently discovered that mesothelial cells are the major driver of tumor stroma enrichment within the CRC PM tumor microenvironment and secreted phosphoprotein 1 (SPP1, also known as osteopontin) is highly expressed by the mesothelial cell-derived stroma [19]. Importantly, in the current study we found SPP1-TWIST1-SPON2 is a key molecular program that mediates tumor-stroma crosstalk and facilitates PM formation. Therefore, SPP1 and SPON2 serve as potential biomarkers and promising therapeutic targets for CRC PM.

## Materials and methods

### Biologic sample preparation

Eligible patients underwent informed consent at the Ohio State University Wexner Medical Center under IRB 2019C0139 and 2019C0196 and at the University of Texas Southwestern Medical Center under IRB STU-2021-1153. Patient specimens were collected from two independent cohorts. The Ohio State University Wexner Medical Center provided samples from patients with peritoneal (*N* = 8) and liver (*N* = 3) metastases. The University of Texas Southwestern Medical Center provided fresh tissue samples, including primary colon tumors (*N* = 6) and omental metastases (*N* = 8). Excess, deidentified tumor specimens were obtained, processed, and entered into the biorepository. Deidentified specimens were utilized to generate protein lysates.

### CRISPR Cas9 knockout of TWIST1 and SPON2

CRISPR-Cas9 knockout of TWIST1 in colorectal cell lines MDST8, MC38, and CT-26 was achieved via electroporation. Guide RNAs were sourced from Integrated DNA Technologies (IDT, Coralville, IA). Human guide RNA sequences for TWIST1 included: TTGCTCAGGCTGTCGTCGGC, GCAAGCGCGGGGGACGCAAG, and CGGGAGTCCGCAGTCTTACG. Mouse guide RNA sequences for TWIST1 were: CACGTTAGCCATGACCCGCT, CGGGAGCCCGCAGTCGTACG, and CGCCGCCCGCGAGATGATGC. Cas9 (Invitrogen, TrueCut) was annealed with guide RNAs and the fluorescent marker ATTO550 (Invitrogen, Waltham, MA). Optimization of electroporation settings was performed using GFP transfection efficiency on the NEON system (Invitrogen, Waltham, MA). Post-optimization, electroporation of MDST8, MC38 and CT-26 cells was conducted. Fluorescently labeled cells were sorted by single-cell isolation and cultured as described. Genomic DNA extraction (Thermo Scientific, Waltham, MA) and PCR amplification of the guide RNA site (forward: CCTCCTCCTCACGTCAGGCCAA and reverse: CTTGCTCAGCTTGTCCGAGGGC) followed by Sanger sequencing were used to evaluate knockout efficiency. Synthego Interference of CRISPR editing was performed to assess knockout efficiency, with Western blotting verifying protein knockout.

Human and mouse SPON2 sgRNA CRISPR/Cas9 All-in-One Lentivector sets were purchased from Applied Biological Materials Incorporated (Richmond, BC, Canada). Mouse SPON2 target sequences were: KO1 GAAAACGTGAGTCTTGCCCT, KO2 TGGAGCCTATCATGGCCAGG, and KO3 CAAACCGATTCTCCCCCCAG. Human SPON2 target sequences were: KO1 CAGATGGACTCTCCCCCAAG, KO2 GGTGATGCTGTATTTGGCCA, and KO3 GCTGTAGTCGGAGCTATGCG. Viral particles were produced using HEK293T cells with the trans-lentiviral packaging system from Horizon Discovery. Transduction was performed on MDST8 and MC38 cells using 4 µg/mL hexadimethrine bromide (Polybrene®) with knockout confirmed by Western blotting in MDST8, MC38, and CT-26 cells.

### RNA-Seq

#### Library generation and sequencing

RNA was isolated using the Qiagen RNeasy Mini Kit (Qiagen, Hilden, Germany). mRNA was purified from total RNA using oligo-dT magnetic beads. After fragmentation, the first-strand cDNA was synthesized using random hexamer primers, followed by the second-strand cDNA synthesis using dUTP for directional libraries. The library was checked using Qubit and RT-PCR for quantification and a bioanalyzer for size distribution detection. Quantified libraries were pooled and sequenced on the Illumina platform NovaSeq 6000 (Illumina, San Diego, CA).

### Data analysis

Raw sequencing reads in FASTQ format were quality trimmed, and adapters were removed using Trim Galore (v0.6.6) (https://www.bioinformatics.babraham.ac.uk/projects/trim_galore/). Trimmed reads were then mapped to the human genome (HG38 assembly) using HISAT2 (v. 2.1.0). Afterward, the mapped reads in SAM format were converted into BAM format, sorted for coordinates, and indexed using samtools (v.1.6) [20]. Sorted BAM files were finally used as input for featureCounts (v.2.0.0) in order to count the mapped reads to the gene coordinates reported in the GTF annotation file downloaded from GENCODE (v.43). Raw counts were scaled using the Reads Per Million (RPM) formula to filter out low-expressed genes prior to normalization and differential expression analysis. Precisely, all the genes whose geometric mean of the RPM was less than one across all samples were removed. Afterward, raw counts of retained genes were log2-transformed and differential expression analysis was performed using the Limma R package. Genes with a |Log2FC| > 0.58 (|Linear FC| > 1.5) and an adjusted *p* < 0.05 (Benjamini-Hochberg correction) were considered differentially expressed. All the analyses have been performed in R (v. 4.2.2) using the RStudio (v. 2022.12.0) framework.

### ChIP-Seq

#### Library generation and sequencing

LoVo and MDST8 cells were cultured, fixed with formaldehyde, lysed, and sonicated. TWIST1 antibody (Abcam ab50887) was used for immunoprecipitation with a bacterial positive control (Abcam). Complexes were captured with Protein G magnetic beads, washed, and DNA purified for library construction and sequencing. Paired-end libraries were generated using Illumina protocols. Libraries were quantified (Qubit3.0), diluted to 1 ng/μL, fragment size checked (Agilent 2100), and effective concentration (>10 nM) determined (Bio-Rad CFX 96 qPCR) to ensure quality. Qualified libraries were sequenced on the Illumina NextSeq 500 platform, and 150-bp paired-end reads were generated.

### Data analysis

Raw sequencing reads in FASTQ format were quality trimmed, and adapters were removed using Trim Galore (https://www.bioinformatics.babraham.ac.uk/projects/trim_galore/). Trimmed reads in FASTQ format were then aligned to the human genome using Bowtie 2 (v.2.4.5) and, following this, they were converted to the BAM format, sorted for coordinates, and indexed using samtools (v.1.6) [20]. Sorted BAM files were finally used as input for MACS2 (v.2.2.7.1) to perform peak calling. Only peaks with a *p* < 0.05 were used for further analysis. Significant peaks were annotated based on their location relative to the TSS of coding-protein genes by CHIPpeakAnno (v.3.30.1) using the genomic coordinates retrieved from ENSEMBL (internal to CHIPpeakAnno)[21]. The visualization of the peaks around the TSS and other genomic features was performed using the ChIPseeker R package (v.1.32.1) [22].

### Animal model

All experiments involving animals were performed in accordance with the protocol approved by the University of Texas Southwestern Medical Center. Syngeneic colorectal PM model was established via intraperitoneal injection utilizing MC38 cells [23]. C57BL/6 mice and SPP1 knockout mice (B6.129S6(Cg)-Spp1tm1Blh/J) were obtained from the Jackson Laboratory (Bar Harbor, ME). Six-week-old C57BL/6 and SPP1 knockout mice were housed in groups of five per cage. Each mouse received an intraperitoneal injection of 50,000 cells from either the TWIST1 or SPON2 knockout or control MC38 cell lines. Mice were monitored until they exhibited severe illness or expired naturally. At 28 days post-tumor injection, the control group mice showed signs warranting euthanasia. Mice were sacrificed, and autopsies were conducted. Peritoneal carcinomatosis index (PCI) was independently assessed with a scoring system equivalent to the PCI as used in humans by three researchers (ACK, MHP, and ZZ) to ensure accurate and unbiased evaluation [24, 25].

### Statistical analysis

Statistical analyses were performed using GraphPad Prism 10. To compare the means of two groups, unpaired Student’s *t* tests were utilized. For comparisons among multiple groups, a one-way ANOVA (for one independent variable) or a two-way ANOVA (for two independent variables) with Tukey’s multiple comparisons test was applied to all pairwise combinations. All statistical tests were two-sided, and a *P* value of <0.05 was considered significant. Mortality rates between groups were compared using log-rank tests, with significance also set at a *P* value of <0.05.

### Ethics statement

All methods were performed in accordance with the relevant guidelines and regulations. Mouse handling and experimental procedures were approved by the Institutional Animal Care and Use Committee (IACUC) at the University of Texas Southwestern Medical Center in accordance with the US National Institutes of Health Guidelines for the Care and Use of Laboratory Animals and the Animal Welfare Act. For the use of human patient samples, the study protocols were approved by the Institutional Review Boards (IRB) at The Ohio State University Wexner Medical Center and the University of Texas Southwestern Medical Center, and informed consent was obtained from all participants.

## Results

### TWIST1 is specifically upregulated in colorectal cancer peritoneal metastases

**Fig. 1:**
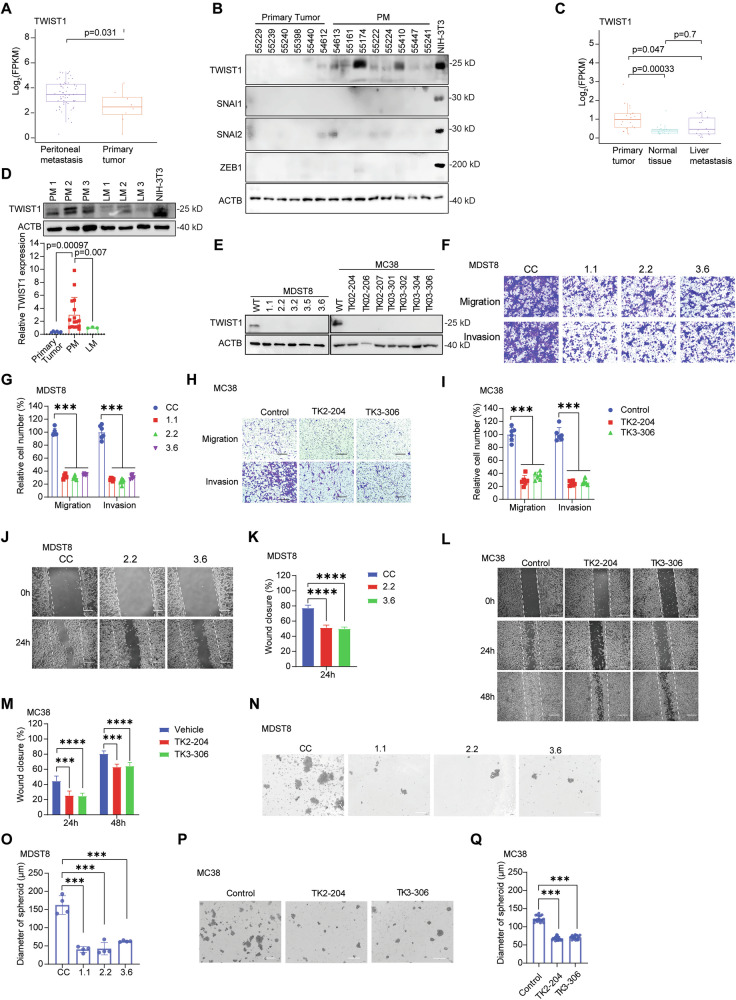
TWIST1 is preferentially expressed in colorectal cancer (CRC) peritoneal metastases (PM) and regulates colon cancer cell migration, invasion, and stemness. **A** Differential expression profiling of CRC PM patient samples reveals upregulation of TWIST1. **B** Western blot of EMT markers (TWIST1, SNAI1, SNAI2, ZEB1) in CRC PM samples, with NIH-3T3 as positive control. **C** TWIST1 expression in CRC liver metastasis (LM) samples shows no difference from normal tissue. **D** Western blot analysis of TWIST1 expression in CRC LM and PM samples. The bottom panel shows a summary of Western blot quantification from (**B**) and the upper (**D**) and Supplemental Fig. S1G. **E** Western blot of TWIST1 in MDST8 and MC38 TWIST1 knockout (KO) cells. Transwell migration and invasion assays for MDST8 (**F**, **G**) and MC38 (**H**, **I**) TWIST1 KO clones. Images (**F**, **H**) and statistics (**G**, **I**). Scale bar: 200 µm. Wound-healing assays for MDST8 (**J**, **K**) and MC38 (**L**, **M**) TWIST1 KO clones. Images (**J**, **L**) and statistics (**K**, **M**). Scale bar: 200 µm. Self-renewal capacity of MDST8 (**N**, **O**) and MC38 (**P**, **Q**) TWIST1 KO clones. Images (**N**, **P**) and statistics (**O**, **Q**). Scale bar: 500 µm. Results are expressed as mean ± SD. *P < 0.05, **P < 0.01, ***P < 0.001, and ****P < 0.0001.

We analyzed a previously published CRC PM dataset, *GSE183202*, to investigate expression patterns of canonical EMT transcription factors (TWIST1, SNAI1, SNAI2, ZEB1) in CRC PM [13]. Differential gene expression profiling comparing CRC PM samples with unpaired primary tumors revealed a significant upregulation of TWIST1 (*p* = 0.031) and SNAI2 (*p* = 0.025), while SNAI1, and ZEB1 revealed no significant changes in expression (Figs. 1A, S1A–F). Western blot analysis of patient-derived specimens further validated TWIST1 was the main EMT transcription factor upregulated in CRC PM compared to primary tumors (Figs. 1B, S1G).

To assess TWIST1 specificity to peritoneal metastases, we examined transcriptomic data from *GSE50760*, which includes CRLM and primary tumors [26], and validated by comparing the protein expression between CRLM and PM tissues. The results showed that expression of TWIST1 was significantly upregulated only in PM (Fig. 1C, D). Collectively, these findings identify TWIST1 as the canonical EMT factor specifically upregulated in CRC PM, suggesting it may contribute to PM.

### TWIST1 promotes migration, invasion, and self-renewal in CRC cells

Next, we performed CRISPR-Cas9-mediated knockout of TWIST1 (*TWIST1*^*KO*^) in the human CMS4 metastatic CRC cell line MDST8 and murine CRC cell lines CT26 and MC38 to elucidate the function of TWIST1 in CRC PM tumorigenesis, (Figs. 1E, S2A) [13, 27]. Additionally, we used shRNA-mediated knockdown in the human non-CMS4 metastatic CRC cell line LoVo to delineate TWIST1-mediated, non-CMS4 specific downstream targets (Figs. 1E, S2B) [27]. Analysis of *TWIST1*^*KO*^ single-cell-derived colonies demonstrated a significant decrease in migration, invasion, and wound closure (Figs. 1F–M, S2C–F), consistent with the established role of TWIST1 in promoting cellular motility and invasive potential essential for the metastatic cascade. In addition, TWIST1 regulates the undifferentiated state in primary CD44^+^ CRC cells demonstrated by enhanced spheroid formation [17]. We challenged *TWIST1*^*KO*^ cells in a three-dimensional (3D) suspension culture to evaluate self-renewal capacity via sphere formation. Notably, *TWIST1*^*KO*^ MDST8 and MC38 cells exhibited reduced sphere formation capacity and spheroid diameter (Fig. 1N–Q). Collectively, these findings indicate that loss of TWIST1 leads to substantial in vitro deficiencies including reductions in migration/invasion and self-renewal, which are critical for distant organ metastasis.

### SPON2 is a direct transcriptional target of TWIST1

**Fig. 2:**
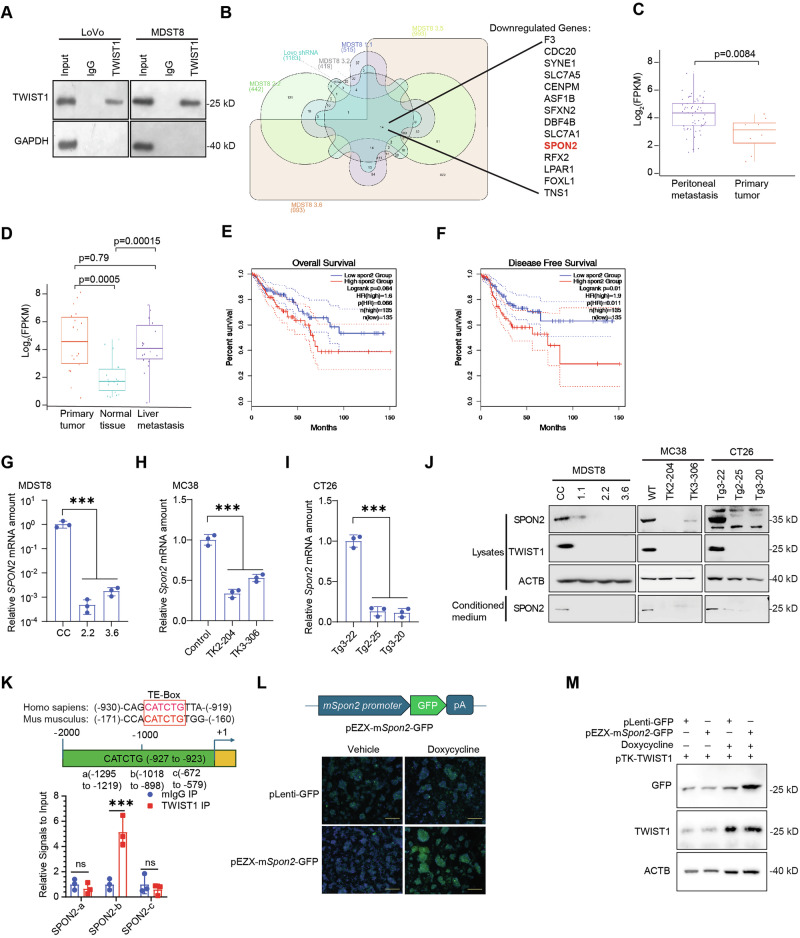
TWIST1 regulates SPON2 expression. **A** Western blot analysis of TWIST1 chromatin immunoprecipitation pull-down in LoVo and MDST8 cells. **B** ChIP-seq and RNA-seq integration in metastatic cell lines, showing TWIST1-bound genes downregulated in TWIST1-deficient LoVo and MDST8 cells; SPON2 highlighted (red). SPON2 expression in CRC PM (**C**) and LM (**D**) patient samples. Kaplan-Meier overall survival (**E**) and disease-free survival (**F**) by SPON2 expression (high vs. low) in TCGA samples. SPON2 mRNA expression in TWIST1 knockout cells, including MDST8 (**G**), CT26 (**H**), and MC38 (**I**). **J** Western blot analysis of SPON2 expression in whole-cell lysates and conditioned medium from TWIST1 knockout MDST8, MC38, and CT26 cells. **K** Top: Schematic of the SPON2 promoter showing the conserved E-box motif (CATCTG) in human and mouse sequences. Bottom: ChIP-qPCR analysis of TWIST1 binding to specific promoter regions (a, b, c) in MDST8 cells. Region “b” contains the predicted E-box. **L** Top: Schematic of the pEZX-mSpon2-GFP promoter reporter construct. Bottom: Representative fluorescence images of cells co-transfected with pEZX-mSpon2-GFP and a Doxycycline-inducible TWIST1 vector (pTK-TWIST1), treated with Vehicle or Doxycycline. **M** Western blot validation of GFP (reporter output) and TWIST1 expression in cells transfected with the indicated combinations of plasmids (pLenti-GFP, pEZX-mSpon2-GFP, pTK-TWIST1) and treated with Doxycycline. Results are expressed as mean ± SD. Statistical significance was determined with ***P < 0.001.

To identify the dysregulated downstream target genes responsible for this phenotype, we performed RNA-seq on *TWIST1*^*KO*^ as well as the shRNA-TWIST1 knockdown cells (Fig. S3). Concurrently, we conducted chromatin immunoprecipitation sequencing (ChIP-Seq) with anti-TWIST1 in wildtype cells to identify direct downstream target genes (Figs. 2A, S3). Through bioinformatic analysis integrating RNA-seq and ChIP-Seq results (Figs. 2B, S3), we identified 27 dysregulated TWIST1 direct target genes. Notably, the following genes were significantly upregulated in *TWIST1*^*KO*^ and shRNA-TWIST1 knockdown cells: *KCNAB2, MIR3142HG, PIK3IP1, PODXL2, SLAIN1, PCDHGC3, MGMT, PDCD4, ACTR3C, TNFRSF21, TNFAIP3, SLC12A2, and PPM1N*. Conversely, the significantly downregulated genes in *TWIST1*^*KO*^ and shRNA-TWIST1 knockdown cells included *F3, CDC20, SYNE1, SLC7A5, CENPM, ASF1B, SFXN2, DBF4B, SLC7A1, RFX2, LPAR1, FOXL1, TNS1, and SPON2* (Figs. 2B, S3).

We further assessed these 27 target genes in CRC PM versus primary tumors to identify clinically relevant, dysregulated direct downstream TWIST1 target genes. *SPON2* was significantly upregulated in CRC PM (*P* = 0.0084) (Fig. 2C). SPON2 is a secreted protein with very limited understanding of its biological function in development and tumorigenesis [28]. Although SPON2 expression has been correlated with CRC, its contribution to metastasis remains unclear. Examination of *SPON2* expression in CRLM compared to primary tumors did not reveal preferential expression (Fig. 2D). TCGA analysis via *GEPIA 2.0* (https://gepia2.cancer-pku.cn/) indicated a trend toward worse overall survival associated with high *SPON2*, with significantly reduced disease-free survival (Fig. 2E, F). To investigate the clinical relationship between TWIST1 and SPON2, we analyzed transcriptomic datasets and observed a significant positive correlation between *TWIST1* and *SPON2* expression in both CRC PM patient samples (Spearman *R* = 0.57) and the large-scale TCGA colorectal cancer cohort (Spearman *R* = 0.77) (Fig. S4A, B). Moreover, *SPON2* levels significantly correlated with disease progression, exhibiting a dramatically increased hazard ratio in advanced stages of CRC (Stage 4 HR = 10.69) in analysis using TIMER 2.0 (http://timer.cistrome.org/) (Fig. S4C). Importantly, analyses of human and murine CRC cell lines with *TWIST1*^*KO*^ demonstrated a significant reduction in mRNA and protein expression of SPON2 (both in lysate and secreted forms), further implicating *SPON2* as a crucial direct target gene of TWIST1 (Fig. 2G–J).

To definitively confirm *SPON2* as a direct transcriptional target, we analyzed the *SPON2* promoter sequence and identified a conserved E-box motif (CATCTG) approximately 1 kb upstream of the transcription start site (Fig. 2K). ChIP-qPCR analysis validated that TWIST1 specifically binds to this E-box-containing region (Region b) compared to adjacent control regions (Fig. 2K). Furthermore, using a *Spon2* promoter-driven GFP reporter system, we observed that Doxycycline-inducible TWIST1 overexpression significantly activated the *Spon2* promoter, resulting in robust GFP expression (Fig. 2L, M). Given the specificity of SPON2 expression in CRC PM and its direct regulation by TWIST1, these data suggest SPON2 may be an important molecule of TWIST1-driven program.

### SPON2 mediates TWIST1-driven metastatic phenotypes

**Fig. 3:**
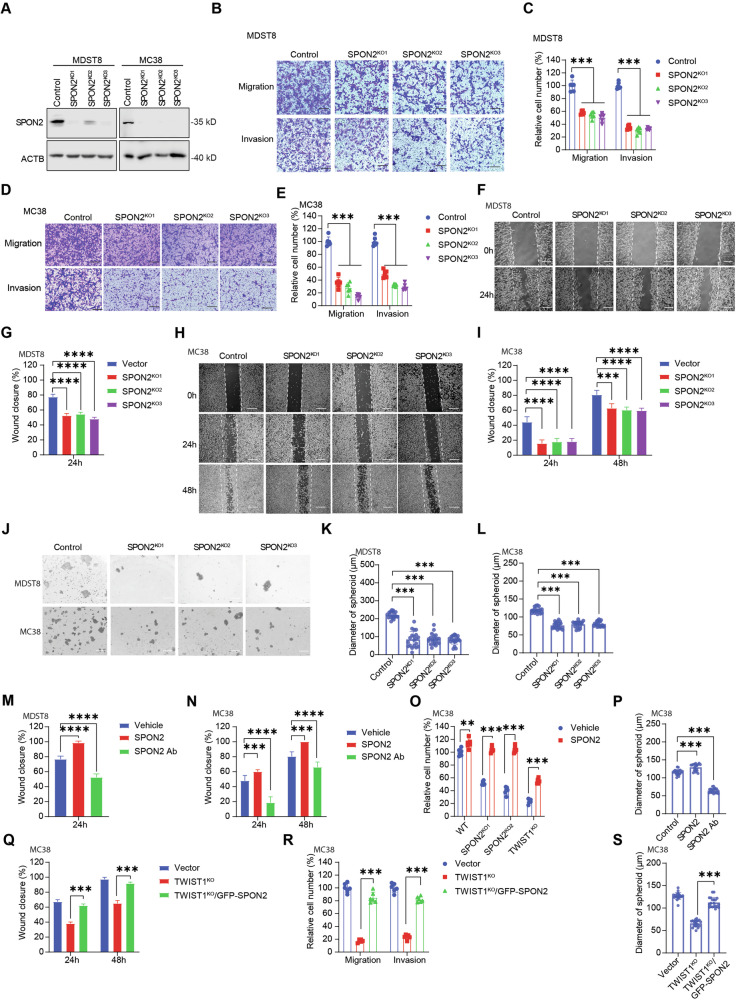
TWIST1-SPON2 cascade regulates colon cancer cell migration, invasion, and stemness. **A** Western blot of SPON2 in MDST8 and MC38 SPON2 KO cells. Transwell migration and invasion assays for MDST8 (**B**, **C**) and MC38 (**D**, **E**) SPON2 KO clones. Representative images (**B**, **D**) and quantitative statistics (**C**, **E**). Scale bar: 200 µm. Wound-healing assay for MDST8 (**F**, **G**) and MC38 (**H**, **I**) cells on Matrigel. Representative images (**F**, **H**) and statistics (**G**, **I**). Scale bar: 200 µm. **J**–**L** Self-renewal assay for MDST8 and MC38 SPON2 KO clones. Representative images (**J**), statistics for MDST8 (**K**) and MC38 (**L**). Scale bar: 500 µm. Wound-healing assay for MDST8 (**M**) and MC38 (**N**) cells on Matrigel with 100 ng/ml SPON2 protein or 1 µg/ml SPON2 antibody. **O** Transwell matrigel invasion assay for MC38 TWIST1 or SPON2 KO cells with 1 mg/ml Matrigel ± 100 ng/ml SPON2 protein, 48 h, 10% FBS. **P** Self-renewal assay for MC38 cells with 100 ng/ml SPON2 protein or 1 µg/ml SPON2 antibody. **Q** Wound-healing assay for MC38 TWIST1 KO cells with GFP-SPON2 overexpression on Matrigel. **R** Transwell migration and invasion assays for MC38 TWIST1 KO cells with GFP-SPON2 overexpression. **S** Self-renewal assay for MC38 TWIST1 KO cells with GFP-SPON2 overexpression. Results are expressed as mean ± SD. *P < 0.05, **P < 0.01, ***P < 0.001, and ****P < 0.0001.

With a goal of better understanding the biologic implication of SPON2, we performed CRISPR-Cas9 knockout of *SPON2* in MDST8 and MC38 metastatic CRC cell lines (Fig. 3A). Consistent with the *TWIST1*^*KO*^ phenotype, *SPON2* knockout (*SPON2*^*KO*^) cells exhibited significant deficiencies in migration, invasion, wound healing, and sphere formation (Figs. 1F–Q, 3B–L). We also conducted neutralization of SPON2 protein with a monoclonal antibody, which resulted in significant and analogous reductions in migration, invasion, wound healing, and sphere formation, highlighting SPON2 as a potential therapeutic target (Figs. 3M–P, S5A–F). Conversely, treatment of MDST8 or MC38 cells with recombinant human SPON2 or murine SPON2, respectively, accelerated wound closure, invasion, and sphere formation in wildtype cells and also rescued the *TWIST1*^*KO*^ and *SPON2*^*KO*^ phenotypes (Fig. 3M–P, S5A–H). Moreover, overexpression of GFP-tagged SPON2 in *TWIST1*^*KO*^ cells rescued the *TWIST1*^*KO*^ phenotype, further solidifying that SPON2 is critical in mediating pro-metastatic cellular processes downstream of TWIST1 (Fig. 3Q–S, S5I–L).

### Stromal SPP1 activates the TWIST1-SPON2 axis via PI3K/AKT signaling

**Fig. 4:**
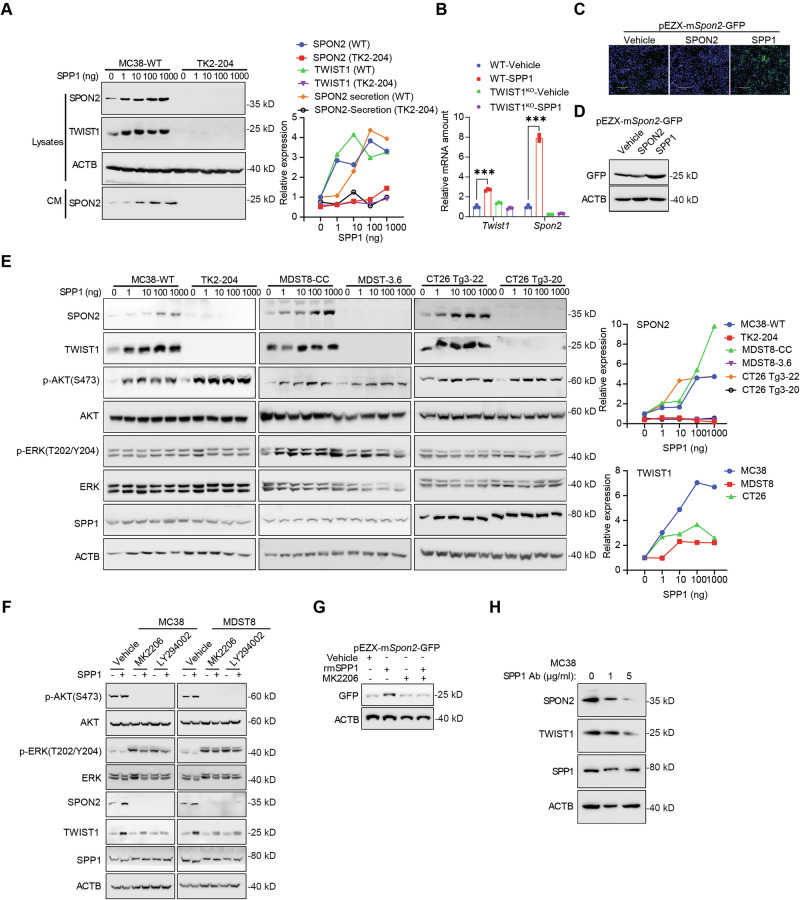
SPP1 enhances the TWIST1-SPON2 cascade. **A** TWIST1 and SPON2 expression in MC38 cell lysates and SPON2 in conditioned medium after 24-h SPP1 pulse-chase at indicated concentrations. Cells were starved for 48 h prior to treatment. Right panel: Quantification of relative protein expression and secretion. **B** RT-qPCR analysis of *Twist1* and *Spon2* mRNA levels in MC38 WT and TWIST1 KO cells treated with vehicle or 100 ng/ml SPP1 for 24 h. **C**, **D** Analysis of *Spon2* promoter activity using a pEZX-m*Spon2*-GFP reporter in MC38 cells treated with Vehicle, SPON2, or SPP1 protein (100 ng/ml) for 24 h. Representative fluorescence images (**C**) and Western blot of GFP expression (**D**) are shown. **E** TWIST1, p-AKT, p-ERK, SPP1, and SPON2 in lysates after 24-h SPP1 pulse-chase at indicated concentrations in MC38, MDST8, and CT26 TWIST1 KO cells starved 48 h. Right panels: Quantification of relative SPON2 and TWIST1 expression. **F** TWIST1, p-AKT, p-ERK, SPP1, and SPON2 in MC38 cells treated with 100 ng/ml SPP1 and 1 µM PI3K/AKT inhibitors (MK2206 or LY294002) for 24 h. **G** Western blot of GFP expression in MC38 cells transfected with the pEZX-m*Spon2*-GFP reporter and treated with 100 ng/ml SPP1 ± 1 µM MK2206. **H** Expression of TWIST1 and SPON2 in MC38 cells treated with 1 or 5 µg/ml SPP1 monoclonal antibody for 24 h. Results are expressed as mean ± SD. *P < 0.05, **P < 0.01, ***P < 0.001, and ****P < 0.0001.

TWIST1 expression, specifically in CRC, is an important regulator of tumorigenesis and metastasis through transcriptional regulation of dedifferentiation of the CD44^+^ CRC population [17]. One of the established ligands for CD44 is secreted phosphoprotein 1 (SPP1). SPP1 is a paracrine factor secreted by cells within the tumor stroma in various cancers including CRC PM [19, 29]. Therefore, we examined whether SPP1 stimulation in tumor cells could induce expression of TWIST1 and subsequent transcription of SPON2. Stimulation of MDST8 and MC38 cells with SPP1 led to a significant dose-dependent induction in *TWIST1* and *SPON2* transcript levels, protein expression with quantification showing a clear linear correlation, and subsequent SPON2 secretion (Fig. 4A, B). Furthermore, qPCR analysis confirmed that SPP1 treatment in WT cells significantly upregulated both *Twist1* and *Spon2* mRNA, an effect that was completely abolished in *TWIST1*^*KO*^ cells (Fig. 4B). We further established stable MC38 cells transfected with pEZX-mSPON2-GFP, a vector with GFP under the control of the murine *Spon2* promoter (Fig. 2L). Stimulation of these cells with recombinant Spp1 protein resulted in enhanced GFP expression, confirming the regulation of *Spon2* gene transcription by Spp1 in cancer cells (Fig. 4C, D).

Prior studies have reported that SPP1 regulates TWIST1 function by mediating signaling through the AKT and ERK pathways in breast cancer [30]. In CRC cells, SPP1 stimulation resulted in increased AKT phosphorylation without affecting ERK phosphorylation (Fig. 4E). This specific signal activation was consistent across multiple cell lines (MC38, MDST8, CT26), where SPP1 treatment dose-dependently increased p-AKT levels alongside TWIST1 and SPON2 upregulation (Fig. 4E, right panels and Fig. S6A). Furthermore, inhibition of the PI3K/AKT pathway utilizing small molecule inhibitors, MK2206 and LY294002, reduced TWIST1 protein levels following SPP1 stimulation, along with concurrent decreases in *SPON2* mRNA and protein expression (Figs. 4F, S6B). The *SPON2* promoter assay confirmed that AKT inhibition with MK2206 blocked SPP1-mediated *SPON2* transcription (Figs. 4G, S6C). Additionally, neutralization of secreted SPP1 with a specific antibody suppressed SPP1-induced expression of TWIST1 and SPON2 proteins (Fig. 4H). To further explore the functional consequences of this signaling axis, we examined the impact of SPP1 on EMT. Our recent study has shown that mesothelial cells are an important source of stromal SPP1 in CRC PM. Therefore, we established an immortalized mesothelial cell line from the mesothelium of mouse omentum (OmenMeso). Co-culture with our proprietary OmenMeso cells or treatment with recombinant SPP1 induced a distinct morphological change in MC38 cells, characterized by a spindle-shaped, mesenchymal phenotype (Fig. S6D). Western blot analysis confirmed this EMT shift, showing upregulation of mesenchymal markers (NCAD, VIM, TWIST1) in both SPP1-treated and OmenMeso co-cultured cells (Fig. S6E). Collectively, these results demonstrate that the TWIST1-SPON2 regulatory cascade is induced through SPP1 in a PI3K/AKT-dependent manner and drives an EMT program conducive to metastasis.

### SPON2 induces mesothelial-to-CAF transition and paracrine SPP1 secretion

**Fig. 5:**
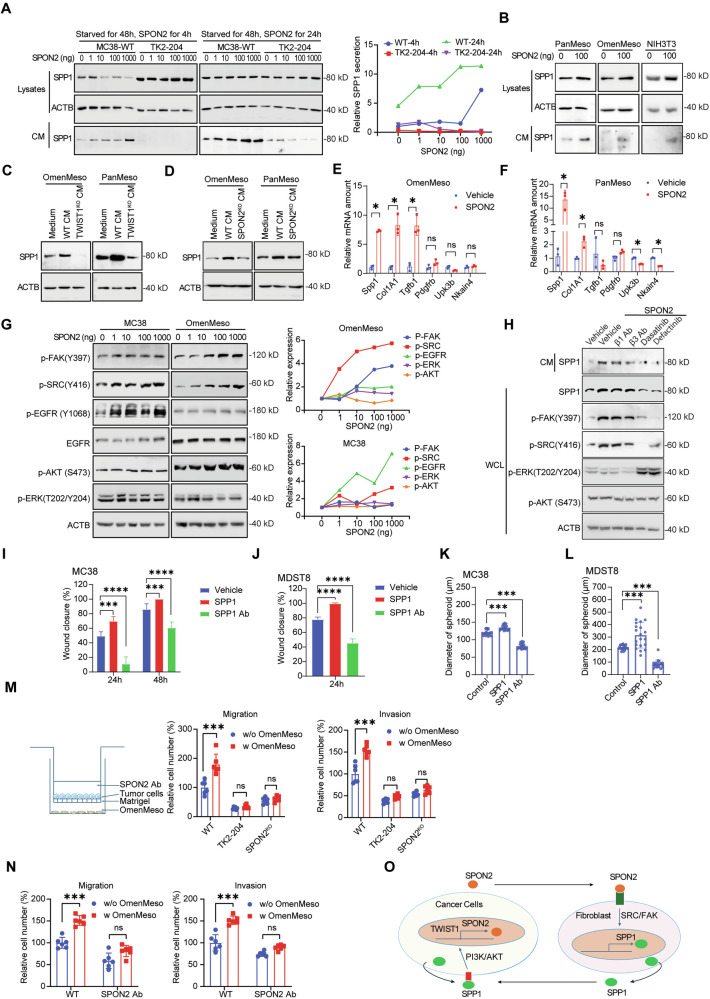
SPON2 regulates SPP1 expression and secretion to promote colon cancer cell migration, invasion, and stemness. **A** SPP1 in MC38 cell lysates and conditioned medium after 4-h or 24-h SPON2 pulse-chase at indicated concentrations, starved 48 h prior to the SPON2 pulse-chase. Right panel: Quantification of relative SPP1 secretion. **B** SPP1 in PanMeso, OmenMeso, and NIH-3T3 cell lysates after 24-h 100 ng/ml SPON2 treatment. SPP1 in PanMeso and OmenMeso cell lysates after incubation with MC38 TWIST1 KO (**C**) and SPON2 KO (**D**) conditioned medium. RT-qPCR analysis of mesothelial/CAF markers (*Spp1, Col1a1, Tgfb1, Pdgfrb, Upk3b, Nkain4*) in OmenMeso (**E**) and PanMeso (**F**) cells treated with 100 ng/ml SPON2 protein for 24 h. **G** Western blot analysis of signaling pathway activation (p-FAK, p-Src, p-EGFR, p-AKT, p-ERK) in MC38 and OmenMeso cells treated with increasing concentrations of SPON2 (0–1000 ng). Right panels: Quantification of relative signaling activation. **H** Western blot analysis of SPP1 secretion and downstream signaling (p-FAK, p-Src, p-ERK, p-AKT) in MC38 cells treated with SPON2 protein in the presence of neutralizing antibodies (β1 Ab eBioHMb1-1, β3 Ab 2C9.G3) or SRC/FAK inhibitors (Dasatinib, Defactinib). Wound-healing assays for MC38 (**I**) and MDST8 (**J**) cells on Matrigel with 100 ng/ml SPP1 protein or 1 µg/ml SPP1 antibody. Self-renewal assay for MC38 (**K**) and MDST8 (**L**) cells with 100 ng/ml SPP1 protein or 1 µg/ml SPP1 antibody. **M** Left: Schematic representation of the transwell co-culture system with OmenMeso cells. Right: Quantification of migration and invasion for MC38 WT, TWIST1 KO (TK2-204), and SPON2 KO cells co-cultured with or without OmenMeso cells. **N** Quantification of migration and invasion for MC38 cells treated with SPON2 neutralizing antibody in the presence or absence of OmenMeso co-culture. **O** Schematic representation of the SPON2-SPP1 feedback loop. Results are expressed as mean ± SD. *P < 0.05, **P < 0.01, ***P < 0.001, and ****P < 0.0001.

We have previously shown in CRC PM, SPP1 can be derived from stromal cells and cancer cells [19]. First, we investigated whether TWIST1-SPON2 regulates the autocrine SPP1 signaling within cancer cells. Notably, comparison of wildtype cells to *TWIST1*^*KO*^ cells revealed a significant decrease in secreted SPP1 protein levels, despite no alteration in cytoplasmic levels (Fig. S7A). Treatment of MC38 cells with recombinant SPON2 protein resulted in a significant increase in secreted SPP1 (Fig. 5A). Conversely, *TWIST1*^*KO*^ cells exhibited suppressed secretion, even in setting of SPON2 stimulation (Fig. 5A), suggesting that the tumor cell-intrinsic SPP1 secretion mechanism may be mediated downstream of TWIST1.

Next, we examined whether tumor-derived secreted SPON2 influences stromal cells, particularly in regulating SPP1 secretion. Our recent study identified mesothelial cells—lining the peritoneal surface—as a major cell type involved in PM. Importantly we also found that mesothelial cells are a major source of stromal SPP1 in CRC PM [31]. Given the importance of tumor-mesothelial interactions in CRC PM progression, we investigated the effect of SPON2 on mesothelial cells [19]. We treated two mesothelial cell lines, PanMeso and OmenMeso—derived from pancreatic and omental mesothelium, respectively—with recombinant SPON2 or cancer cell-conditioned medium (CM). Treatment with SPON2 or CM from wildtype MC38 cells significantly increased SPP1 levels in mesothelial cells compared to treatment with CM from *TWIST1*^*KO*^ cells (Fig. 5B, C). Additionally, CM from *SPON2*^*KO*^ cells resulted in reduced *SPP1* expression, suggesting SPON2 as an upstream regulator of *SPP1* in stromal cells (Fig. 5D). We then transfected OmenMeso cells with a *Spp1* promoter-driven *mCherry* reporter (pEZX-mSpp1-mCherry). Upon stimulation with Spon2 protein the mesothelial cells showed enhanced mCherry expression, validating that Spon2 directly regulates Spp1 transcription in stromal cells (Fig. S7B–D). The distinct responses of cancer and stromal cells to SPON2, where SPON2 enhances cancer cell SPP1 secretion, while upregulating both intracellular and secreted SPP1 in stromal cells, may reflect cell-type-specific signaling pathways or receptor expression profiles.

To further investigate whether tumor-secreted SPON2 mediates plasticity of the mesothelial cells, we performed quantitative PCR and western blot analysis on a panel of fibroblast differentiation markers in PanMeso and OmenMeso cells following treatment with recombinant mSPON2. The results demonstrated that SPON2 significantly promoted the upregulation of genes including *Col1a1, Tgfb1, Pdgfrb, and Spp1*, as well as protein expression of CAF markers Col1a1 (Fig. S7E), suggesting that SPON2 can drive the differentiation of PanMeso and OmenMeso into cancer-associated fibroblasts (Fig. 5E, F). This transition was further supported by morphological changes observed in co-culture (Fig. S7F). Such novel finding highlights that tumor-derived SPON2 induces SPP1 secretion in mesothelial cells within the tumor microenvironment and is also critical in stimulation of mesothelial cells during PM.

### SPON2 signaling in mesothelial cells is Src/FAK-dependent and integrin-independent

The importance of SPON2 in metastatic CRC has been previously established, with various receptors, including α5β1, proposed to mediate the downstream effects of SPON2 [28]. While the precise mechanism by which SPON2 induces SPP1 secretion remains unclear, we hypothesized it might involve integrin-mediated signaling, as described for other matricellular proteins [32]. To elucidate the precise mechanism by which SPON2 induces SPP1 secretion, we analyzed integrin downstream signaling pathways. Treatment with SPON2 induced a dose-dependent phosphorylation of Egfr (Y1068) in MC38 cancer cells, whereas it induced phosphorylation of Fak (Y397) and Src (Y416) in OmenMeso mesothelial cells (Fig. 5G), suggesting distinct SPON2 signaling pathways in tumor versus mesothelial cells. Crucially, however, the SPON2-induced upregulation of SPP1 secretion and downstream signaling activation (p-Fak, p-Src) were not affected by neutralizing antibodies against Integrin β1 or β3, but were abrogated by the Src inhibitor Dasatinib and the FAK inhibitor Defactinib (Fig. 5H). These results definitively identify that SPON2 promotes SPP1 secretion and signaling activation via a Src/Fak-dependent pathway, but independent of Integrin β1 or β3, implying that the specific SPON2 receptor on mesothelial cells remains to be identified.

To assess the functional consequences of SPON2-induced SPP1 expression, we treated wild-type MC38 and MDST8 cells with recombinant SPP1 protein and observed a significant increase in wound closure, migration, invasion, and sphere formation (Figs. 5I–L and S7G–J). Notably, neutralization of SPP1 markedly reduced these pro-tumorigenic properties, demonstrating that SPP1 is a key mediator of tumor cell aggressiveness (Figs. 5I–L and S7G–J). To confirm the role of the mesothelium in this process, we utilized a transwell co-culture system (Figs. 5M, S7E). Co-culture with OmenMeso cells significantly enhanced the migration and invasion of wild-type MC38 cells; however, this enhancement was abolished in *Twist1*^*KO*^ and *Spon2*^*KO*^ cells (Figs. 5M, S7K). Furthermore, treatment with a Spon2 neutralizing antibody effectively blocked the co-culture-induced migration and invasion (Figs. 5N, S7L). These findings further support that the TWIST1-SPON2 axis drives SPP1 expression and secretion in stromal cells, thereby establishing a pro-tumorigenic microenvironment that facilitates CRC metastasis. Collectively, our results highlight SPON2 as a pivotal regulator of stromal remodeling and tumor progression, with potential implications for therapeutic targeting of the TWIST1-SPON2-SPP1 signaling axis in metastatic CRC.

Furthermore, SPP1-induced upregulation of SPON2 was found to be TWIST1-dependent, indicating the presence of a positive feedback loop that reinforces TWIST1-SPON2 signaling and sustains a pro-metastatic tumor microenvironment in CRC (Fig. 4A, B). This suggests that the TWIST1-SPON2 axis actively drives SPP1 expression and secretion in stromal cells, leading to an accumulation of SPP1 within the tumor microenvironment. This enriched SPP1 pool enhances PI3K/AKT-driven TWIST1-SPON2 signaling in cancer cells, ultimately promoting cancer cell invasion, enrichment of stem-like properties, and metastasis (Fig. 5O).

### The SPP1-TWIST1-SPON2 axis drives peritoneal carcinomatosis and immune exclusion in vivo

**Fig. 6:**
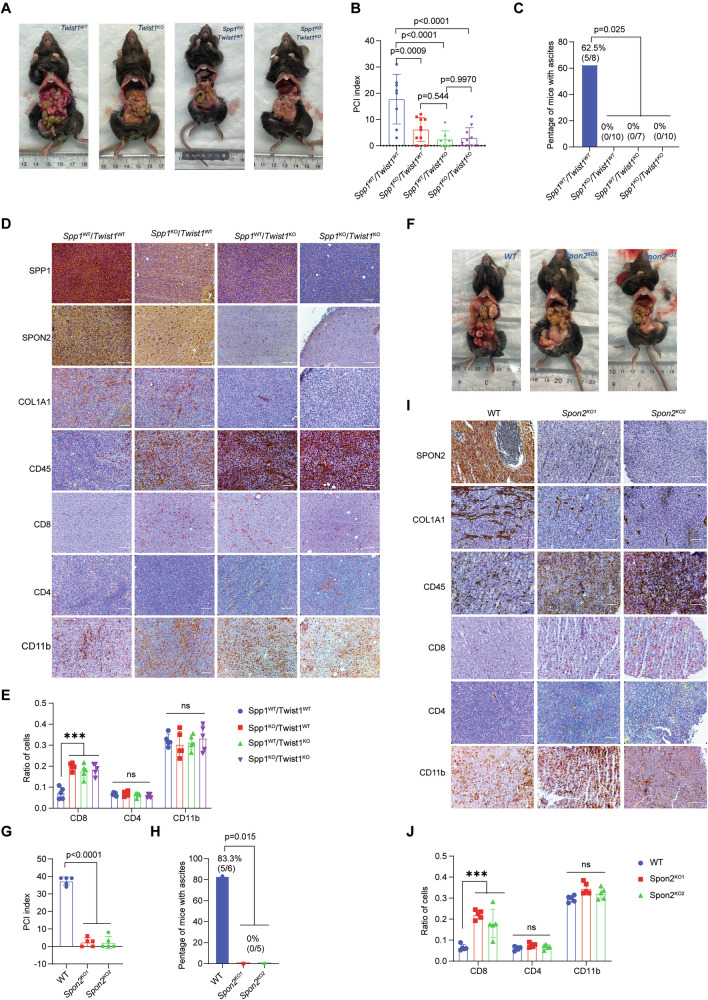
The TWIST1-SPON2-SPP1 cascade regulates peritoneal metastasis. **A** Representative images of peritoneal metastases in mice following intraperitoneal injection of MC38 cells. C57BL/6J mice, with or without *Spp1* gene knockout, were injected with 5 × 10⁴ MC38 cells, with or without *Twist1* knockout, imaged at 28 days. Peritoneal carcinomatosis index (PCI) (**B**) and incidence of ascites (**C**) of mice that underwent intraperitoneal injection of MC38 *Twist1* knockout cells. **D** Immunohistochemical (IHC) staining of SPP1, SPON2, COL1A1, CD45, CD8, CD4, and CD11b in tumor tissue sections from the indicated groups. Scale bar: 200 µm. **E** Quantification of immune cell infiltration (ratio of CD8+, CD4+, and CD11b+ cells) in tumor tissues based on IHC staining from (**D**). **F** Representative images of peritoneal metastases in mice following intraperitoneal injection of MC38 cells with *Spon2* knockout, imaged at 28 days. Peritoneal carcinomatosis index (PCI) (**G**) and incidence of ascites (**H**) of mice that underwent intraperitoneal injection of MC38 *Spon2* knockout cells. **I** Immunohistochemical staining of SPP1, SPON2, COL1A1, CD45, CD8, CD4, and CD11b in tissue sections of MC38 peritoneal metastases. Scale bar: 200 µm. **J** Quantification of immune cell infiltration (ratio of CD8+, CD4+, and CD11b+ cells) in tumor tissues based on IHC staining from (**I**). Results are expressed as mean ± SD. *P < 0.05, **P < 0.01, ***P < 0.001, and ****P < 0.0001.

To assess the contribution of a SPP1-TWIST1-SPON2 axis in CRC PM, we utilized a syngeneic model. We established a syngeneic intraperitoneal PM model using wild-type (WT) *and* Spp1*-deficient (Spp1*^*-/-*^*)* mice injected intraperitoneally with 50,000 MC38-WT or MC38-*Twist1KO* cells. After 28 days, we assessed peritoneal tumor burden using the PCI and evaluated ascites formation [33, 34]. In WT mice injected with MC38-WT cells, the highest tumor burden was observed, as reflected by significantly elevated PCI scores (Figs. 6A, B, and S8A). Additionally, these mice exhibited significant ascites formation, occurring in 62.5% of the control group compared to complete absence (0%) in the knockout groups (Fig. 6C). Interestingly, depletion of stromal SPP1 or tumor-intrinsic TWIST1 led to a significant reduction in Spp1, Spon2, and Col1a1 expression within the tumor, while concurrently increasing *Cd45* expressing cells (Figs. 6D and S8B–E). Detailed immunophenotyping revealed that this increase in immune infiltration was driven specifically by CD8+ cytotoxic T cells, which were significantly enriched in the tumors of the intervention groups, whereas CD4 + T cell and CD11b+ myeloid cell populations remained largely unchanged (Fig. 6D, E). These findings suggest that the SPP1-TWIST1-SPON2 signaling cascade likely contributes to the development of a microenvironment that promotes CRC PM. Additionally, we assessed tumor burden using PCI scores and ascites incidence in mice injected with 50,000 MC38-WT or MC38-*Spon2*^*KO*^ cells to further investigate the impact of SPON2 on tumor progression. Loss of *Spon2* in MC38 cells resulted in a significant reduction in PCI scores, ascites formation, and tumor stroma (Figs. 6F–H and S8F–G). Conversely, *Spon2* depletion led to an increase in CD45-positive immune cell infiltration. Consistent with the *Twist1/Spp1* depletion models, this immune infiltration in *Spon2*-deficient tumors was characterized by a specific and significant upregulation of CD8+ T cells, with no significant differences observed in CD4+ or CD11b+ populations (Fig. 6I, J), suggesting that Spon2 contributes to the establishment of a fibrotic and immune suppressed tumor microenvironment (Fig. 6H). Collectively, these findings demonstrate that tumor-intrinsic TWIST1-SPON2 signaling is a key driver of CRC PM by promoting stroma-derived SPP1 secretion. This axis fosters a pro-tumorigenic microenvironment characterized by enhanced fibrosis and suppression of cytotoxic T cell immunity, highlighting its potential as a therapeutic target in metastatic CRC.

## Discussion

Despite the high global incidence of CRC PM, there remains a significant gap in knowledge of the molecular underpinnings. This lack of understanding contributes to rapid disease progression, frequent resistance to standard systemic therapies, and poor patient prognosis due to limited therapeutic options. Prior molecular profiling of CRC PM demonstrated an enrichment of the CMS4/mesenchymal signature, which is associated with aggressive tumor behavior and poor outcomes [12, 35]. The present study expands on this observation through genetic and molecular characterization, which identified *TWIST1* and its direct downstream target, *SPON2*, to be specifically upregulated in CRC PM. Moreover, the activation of SPP1-TWIST1-SPON2 cascade and the dissection of the axis allowed for the elucidation of molecular crosstalk within the tumor microenvironment between the tumor epithelium and stroma, in vitro and in vivo. Furthermore, the study identified the constituents of this signaling axis as a potential novel biomarkers and innovative therapeutic targets for CRC PM.

Emerging evidence highlights that TWIST1 is crucial in CRC progression, particularly through activation of downstream genes that contribute to metastasis and therapy resistance [16, 36–38]. The correlation between high TWIST1 expression and advanced tumor stage, lymph node metastasis, and poor survival rates underscores its potential as a prognostic biomarker [15, 39, 40]. Moreover, TWIST1 has been implicated in the regulation of cancer stem cell properties, which contribute to tumor initiation, recurrence, and resistance to chemotherapeutic agents such as 5-fluorouracil and oxaliplatin [41]. This multifaceted contribution to CRC progression and specific upregulation within CRC PM is demonstrated in the downregulation of TWIST1 resulting in depletion of invasion properties and self-renewal capacity [17].

Notwithstanding the importance of TWIST1 in CRC PM, *SPON2* was defined as a direct downstream target responsible for the tumor phenotype. We definitively validated this direct regulation through the identification of a conserved E-box motif in the *SPON2* promoter and ChIP-qPCR confirmation of TWIST1 binding. In CRC, high SPON2 expression correlated significantly with worse disease-free survival and with advanced cancer stages (HR > 10 in Stage 4). Although SPON2 has been previously reported to mediate migration and invasion in CRC and serve as a prognostic marker for liver metastasis [42], our data reveals a critical distinction. Our transcriptomic analysis of paired samples demonstrated that *TWIST1* and *SPON2* are specifically upregulated in PM but NOT in CRLM (Figs. 1C, D, 2D). Given Lenos et al.’s [13] demonstration that CMS4 enrichment in CRC PM and predominantly CMS2 phenotype (>60%) with the remaining fraction being almost exclusively CMS4 in CRC LM, the observation by Schmid et al. [42], likely provides a mechanism for this specific CMS4 subgroup within hepatic metastases. Moreover, this suggests a context-specific role for this axis in the peritoneal niche, distinct from the dominant process in liver metastasis. *SPON2* encodes a secreted protein with limited known cell intrinsic and extrinsic functions. *SPON2*^*KO*^ cells phenocopied *TWIST1*^*KO*^ cells in assessment of intrinsic in vitro metastatic potential, while addition of recombinant SPON2 or overexpressed SPON2 resulted in a rescue of these deficient phenotypes. Importantly, antibody-mediated neutralization of SPON2 in cancer cells resulted in depletion of metastatic phenotype, analogous to *SPON2*^*KO*^. In a disease-state devoid of efficacious therapeutic options, SPON2 may potentially serve as a novel biomarker and therapeutic target.

The cellular and molecular composition of tumor microenvironment serves to foster the malignant potential of specific cells to gain metastatic potential and exacerbate therapeutic resistance [43–45]. The peritoneum is a tissue that consists of various stromal cells, including mesothelial cells, fibroblasts, endothelial cells and immune cells. Our recent study has shown that mesothelial cells can differentiate into a specific CAF population, known as antigen-presenting cancer-associated fibroblasts (apCAFs), contribute to cancer progression [31]. CAFs can interact with tumor epithelium through paracrine signaling, such as TGF-β, to support aggressive cancer phenotypes [17]. More importantly, CAF-derived SPP1 was recently identified as a critical factor in maintaining a pro-tumor microenvironment and stemness of cancer cells through CD44 [29]. Additionally, SPP1 expression in tumor-associated macrophages has been reported to promote tumor progression and immune suppression in CRC [46]. However, our investigation identified mesothelial cells as an important source of stromal SPP1 in CRC PM. In turn, SPP1 stimulation of tumor cells through CD44 and activated the PI3K/AKT signaling cascade to induce TWIST1-SPON2 transcriptional axis in tumor cells to enhance the metastatic potential. This novel observation highlights the important contribution of TME for CRC PM tumorigenesis

Lastly, SPON2, as a secreted factor, likely exerts its biologic function through paracrine-mediated regulation of not only tumor cells but also within the TME. Stimulation with recombinant SPON2 or conditioned media containing SPON2 demonstrated increased SPP1 expression and secretion. Notably, SPON2 differentially regulates SPP1 in cancer and stromal cells: in cancer cells, SPON2 only enhancing secretion, likely through TWIST1-dependent mechanisms, whereas in stromal cells, such as mesothelial cells, SPON2 upregulates both intracellular and secreted SPP1, via direct transcriptional activation as evidenced by promoter reporter assays. These distinct responses may arise from cell-type-specific signaling pathways or differential receptor engagement, though the precise mechanisms, including the specific receptors mediating SPON2 effects, remain under investigation. More importantly, SPON2 stimulation initiated the mesothelial differentiation demonstrating a novel paracrine signaling circuit between tumor cells and stroma that drives the CRC PM tumorigenesis. We found that SPON2 activates EGFR in tumor cells but triggers FAK/SRC signaling in mesothelial cells. Crucially, our inhibitor studies revealed that Spon2-mediated Spp1 secretion and signaling are dependent on Src and Fak activity but, surprisingly, are independent of Integrin β1 or β3, suggesting the existence of an alternative, yet-to-be-identified SPON2 receptor on mesothelial cells.

Despite the limitation of in vivo intraperitoneal injection models and non-existent transgenic CRC PM models, our in vivo experiment further established this important novel circuit in CRC PM tumorigenesis and subsequent ascites production. Ascites production is frequently observed in PM and is a significant contributor of poor patient outcome due to abdominal pain, anorexia, nausea, and cachexia [47]. Commonly accepted hypothesis is that the tumor cells were primarily responsible for ascites production. Through our transgenic experiment, the stromal SPP1 secretion and subsequent SPON2 expression are likely responsible for ascites production. Following complete abolition of ascites in stromal SPP1 knockout mice, we hypothesize that mesothelial cells and/or stroma are the main activator of ascites production rather than the tumor epithelium. Furthermore, our immune profiling revealed a critical role for the SPP1-TWIST1-SPON2 axis in immune evasion. Recently, SPON2 was reported to promote macrophage polarization toward an immunosuppressive M2 phenotype in osteosarcoma [48]. Interestingly, while we observed a significant immune exclusion phenotype, depletion of any component of this axis (SPP1, TWIST1, or SPON2) resulted in a significant infiltration of CD8+ cytotoxic T cells into the tumor, while CD4+ and CD11b+ populations remained largely unaffected. This robust enhancement of CD8+ T-cell infiltration following genetic ablation of stromal SPP1 or tumor TWIST1 is a particularly intriguing finding with significant biological and clinical implications. SPP1 is increasingly recognized as a potent immunosuppressive cytokine that helps construct a physical and biochemical barrier against T-cell trafficking, often by promoting a dense fibrotic matrix and directly suppressing T-cell activation [19, 46, 49]. By disrupting the SPP1-TWIST1-SPON2 paracrine loop, this fibrotic and immunosuppressive barrier is likely compromised, effectively transforming a “cold,” immune-excluded peritoneal microenvironment into a “hot,” T-cell-inflamed tumor bed within the peritoneal microenvironment. This indicates that in the context of CRC PM, the TWIST1-SPON2 axis primarily drives the exclusion of cytotoxic T cells rather than macrophage modulation, thereby fostering a fibrotic and immune-suppressed niche permissible for metastasis.

In conclusion, our findings identify a novel SPP1-TWIST1-SPON2 axis that plays a critical role in the progression of CRC PM. This axis functions as both a key regulatory mechanism and a driver of tumor invasion and metastasis, fibrosis, and immune exclusion within the peritoneal tumor microenvironment. Given its pivotal role, the SPP1-TWIST1-SPON2 axis holds significant potential as a biomarker for disease progression, an effector of metastatic signaling, and a promising therapeutic target for CRC PM intervention.

## Supplementary information

Supplemental Materials

## Data Availability

The datasets (GSE183202 and GSE50760) analyzed during the current study are available in the Gene Expression Omnibus (RRID:SCR_005012) under its identifier. RNA-seq and ChIP-seq data generated in this study have been deposited in the Gene Expression Omnibus (GEO) under accession number GSE319741. All data generated or analyzed during this study are available from the corresponding author on reasonable request.
