# Supplementary material for: TWIST1 mediated transcriptional activation of SPON2 drives colorectal cancer peritoneal metastasis through stromal cell signaling network
Source: Oncogene. 2026 Apr 3;45(17):1613–26. doi: 10.1038/s41388-026-03743-7 (PMC13099403; doi:10.1038/s41388-026-03743-7)

Supplemental Materials

TWIST1 mediated transcriptional activation of *SPON2* drives colorectal peritoneal metastasis through stromal cell signaling network

Zhuan Zhou[1], Alessandro La Ferlita[2], Manoj H. Palavalli[3], Xiongfeng Chen[1], Lauren Tyler[1], Aslam Ejaz[4], Joal Beane[3], Patricio M. Polanco[1], Huocong Huang[1, §], Alex C. Kim[1,5, §]

Contents

1. **Key resources**
2. **Supplemental Materials and Methods**
3. **Supplemental Figures**
4. **Uncropped gels**

1. Key resources

REAGENT OR RESOURCE	SOURCE	IDENTIFIER
**Antibodies**		
SPON2	Thermo Fisher Scientific	20513-1-AP
SPON2	Thermo Fisher Scientific	PA5-106790
SPP1	InVivoMAb	BE0382
TWIST1	Abcam	50887
p-AKT (Ser473)	Cell signaling technology	3700S
AKT	Cell signaling technology	9272S
p44/42 MAPK (Erk1/2) (Thr202/Tyr204)	Cell signaling technology	4370S
p44/42 MAPK (Erk1/2)	Cell signaling technology	4695S
ACTIN	Cell signaling technology	3700S
CD45	Cell signaling technology	70257S
COL1A1	Cell signaling technology	72026S
SNAI1	Cell signaling technology	3879S
SNAI2	Cell signaling technology	9585S
ZEB1	Cell signaling technology	3396S
GAPDH	Cell signaling technology	2118S
CD61	LIFE TECHNOLOGIES	16-0611-82
CD29	LIFE TECHNOLOGIES	14-0291-82
CD4	Cell signaling	25229T
CD8	Cell signaling	98941T
CD11b	Abcam	ab133357
Vimentin	Cell signaling technology	5741T
NCAD	Cell signaling technology	13116T
p-SRC (Tyr416)	Cell signaling technology	2101S
p-FAK (Tyr397)	Cell signaling technology	3283S
Alexa 488-anti-mouse	Cell signaling technology	4408S
Alexa 488-anti-rabbit	Cell signaling technology	8889S

Anti-rabbit IgG, HRP-linked	Cell signaling technology	7074S
Anti-mouse IgG, HRP-linked	Cell signaling technology	7076S
ImmPRESS HRP Goat anti-Mouse	VectorLabs	MP7452
ImmPRESS HRP Goat anti-Rabbit	VectorLabs	MP7451
**Chemicals, peptides, and recombinant proteins**		
Dasatinib	MedChemExpress	HY-10181
Defactinib	MedChemExpress	HY-12289
MK-2206	MedChemExpress	HY-108232
G418	Thermo Fisher scientific	MT30234CR
Puromycin	Thermo Fisher Scientific	A1113802
RIPA lysis buffer	Cell Signaling Technology	9806
DMEM	Sigma-Aldrich	D6429
Phosphate-buffered saline	Thermo Fisher Scientific	AM9625
TBST	Cell Signaling Technology	9997S
Triton X-100	Sigma-Aldrich	93443
DTT	Sigma-Aldrich	D0632
Paraformaldehyde	Thermo Fisher Scientific	AAJ19943K2
Protease inhibitor cocktail	Thermo Fisher Scientific	78430
Phosphatase inhibitors	Thermo Fisher Scientific	78420
Matrigel® Growth Factor Reduced	Corning	356231
Recombinant Human R-Spondin 2 Protein	R&D Systems	3266-RS-025/CF
Recombinant Mouse R-Spondin 2 Protein	R&D Systems	6946-RS-025
Recombinant Mouse Osteopontin/OPN Protein	R&D Systems	441-OP-050
Mouse R-Spondin 2 Antibody	R&D Systems	MAB32661
**Critical commercial assays**		
BCA Assay Kit	Thermo Fisher Scientific	23225

Betazoid DAB Chromogen	Fisher Scientific	50-823-73
RNeasy Plus Mini Kit	QIAGEN	74136
iScript cDNA Synthesis Kit	Bio-Rad	170-8891
Plasmid Midi PREP	Qiagen	12143
SuperSignal West Pico Sensitivity Substrate	Thermo Fisher Scientific	34080
**Experimental models: cell lines**		
LoVo	ATCC	CCL-229
HEK293T	ATCC	CRL-3216
LoVo	ATCC	CRL-1469
MC38	Sigma	SCC172
CT-26	ATCC	CRL-2638
3T3	ATCC	CRL-1658
L-WRN	ATCC	CRL-3276
**Recombinant DNA**		
GFP-SPON2	Sino Biological INC	HG12452-ACG
pTK-TWIST1	Addgene	Plasmid #36977
SPON2 sgRNA CRISPR/Cas9 All-in-One Lentivector set (Human)	Applied Biological Materials (abm)	45366111
Spon2 sgRNA CRISPR/Cas9 All-in-One Lentivector set (Mouse)	Applied Biological Materials (abm)	45366114
Promoter reporter clone for Mouse Spon2 (NM_133903	GeneCopoeia	MPRM64354-LvPF02
Promoter reporter clone for Mouse Spp1 (NM_009263	GeneCopoeia	MPRM80524-LvPM02
**Primers**		
SPON2-a-F AAACATTTGAGAAGCACGGAGG	Sigma-Aldrich	This paper
SPON2-a-R AGCCACTCAATGCAGTATTTAGAC	Sigma-Aldrich	This paper

SPON2-b-F CATTCCCGAGCTCCTTCCTC	Sigma-Aldrich	This paper
SPON2-b-R CCCAGGGGCTCTAGGCTT	Sigma-Aldrich	This paper
SPON2-c-F GCCATGCCAGGAAAGAGACT	Sigma-Aldrich	This paper
SPON2-c-R TACATGCTGGAAGTGGCTGG	Sigma-Aldrich	This paper
hSPP1-F CGAGGTGATAGTGTGGTTTATGG	Sigma-Aldrich	This paper
hSPP1-R GCACCATTCAACTCCTCGCTTTC	Sigma-Aldrich	This paper
mSPP1-F GCTTGGCTTATGGACTGAGGTC	Sigma-Aldrich	This paper
mSPP1-R CCTTAGACTCACCGCTCTTCATG	Sigma-Aldrich	This paper
hSPON2-F ACAGCATCACCTTCACGGGCAA	Sigma-Aldrich	This paper
hSPON2-R CTGACGTACTGGTTCTTCCTCC	Sigma-Aldrich	This paper
mSPON2-F CGACAGTGGTTTCACCTTCTCC	Sigma-Aldrich	This paper

mSPON2-R AGGACTTGAGGCGTGGGTAGTA	Sigma-Aldrich	This paper
	Sigma-Aldrich	This paper
**Software and algorithms**		
Image Lab software 6.0	Bio-Rad	https://www.bio-rad.com/en-us/product/image-lab-software?ID=KRE6P5E8Z
CFX Manager software 2.0	Bio-Rad	https://www.bio-rad.com/en-us/sku/1845000-cfx-manager-software?ID=1845000
GraphPad Prism 9	GraphPad Software	https://www.graphpad.com/scientific-software/prism/
ImageJ (Fiji 2.9.0)	NIH	https://imagej.net/downloads

2. **Supplemental Methods**

Cell culture

The cell lines LoVo, CT-26, 3T3, L-WRN, and 293FT were obtained from the American Type Culture Collection (ATCC, Manassas, Virginia, USA). MC38 and MDST8 were procured from Sigma-Aldrich (St. Louis, MO, USA). LoVo (CMS2), CT26, and MC38

(CMS4-like) were selected to represent non-CMS4 and CMS4-like phenotypes, respectively, while MDST8 (CMS4) was used as a PM-specific model. Cell lines were grown at 37°C in a humidified atmosphere containing 5% CO2. Cultures were maintained in either Dulbecco Modified Eagle Medium (DMEM) (Corning, Corning, NY, USA), F-12 (Gibco, Waltham, MA, USA), or RPMI 1640 (Gibco, Waltham, MA, USA) containing 10% heat-inactivated fetal bovine serum (Sigma-Aldrich, St. Louis, MO, USA), 15 mM HEPES, L-glutamine, and 1% penicillin-streptomycin (Gibco, Waltham, MA, USA). Subculturing of 80%–90% confluent cells was routinely performed using trypsin-EDTA solution (0.25% trypsin and 0.53 mM EDTA).

Pancreatic mesothelial (PanMeso) cells were established and cultured as previously reported [20]. Omental mesothelial (OmenMeso) cells were established as follows. In brief, mesothelium was harvested from the normal omentum of immortomice (Taconic Biosciences) under a dissecting microscope. The omental mesothelium was seeded onto a tissue culture dish and cultured at 33°C. Expanded cells were collected and named OmenMeso cells. PanMeso and OmenMeso cells were cultured in mesothelial cell media (medium 199 (Gibco/Thermo), 10% FBS, 1% penicillin-streptomycin (Gibco/Thermo), 3.3 nM mouse epidermal growth factor (Biolegend), 400 nM hydrocortisone (MilliporeSigma), 870 nM zinc-free bovine insulin (MilliporeSigma), 20 mM HEPES (Thermo Fisher Scientific)), maintained at 33°C, washed with PBS three times, and cultured in fresh mesothelial cell media at 37°C for three days before any experiments to inactivate immortalization traits. Cells were regularly tested for mycoplasma infection and confirmed to be mycoplasma-free using the e-Myco kit (Boca Scientific).

shRNA knockdown of TWIST1

Lentiviral short hairpin RNA constructs targeting TWIST1 were obtained from Horizon Discovery (Waterbeach, UK). The target sequences were: AGGAAGAGCCAGACCGGCA, TGTCCGCGTCCCACTAGCA, and GCGGCCAGGTACATCGACT. Viral particles were produced using HEK293T cells with the trans-lentiviral packaging system from Horizon Discovery. Transduction was performed on LoVo cells using 4 μg/mL hexadimethrine bromide (Polybrene). Viral induction was carried out for 8 hours before the media was replaced. Knockdown efficiency was assessed at the mRNA level via qRT-PCR 5 days post-transduction using TaqMan probes (Thermo Fisher Scientific, assay ID# HS04989912_s1) for TWIST1 (Thermo Fisher, Waltham, MA, USA).

Plasmids

The mouse *Spp1* (NM_009263) promoter mCherry reporter (#MPRM80524-LvPM02; GeneCopoeia) and the mouse *Spon2* (NM_133903) promoter GFP reporter (#MPRM64354-LvPF02; GeneCopoeia) were purchased from GeneCopoeia. The GFP-SPON2 expression plasmid (#HG12452-ACG) was obtained from Sino Biological Inc. Plasmids were transfected via Lipofectamine 3000 (Thermo Fisher) to study promoter activity and protein expression. The doxycycline-inducible pTK-TWIST1 (Addgene plasmid #36977) plasmid was obtained from Addgene.

Western blotting

Proteins were extracted using RIPA buffer (Invitrogen, Waltham, MA, USA), and protein concentrations were determined using a Pierce BCA kit (Thermo Fisher, Waltham, MA, USA). Equal amounts of protein were separated on polyacrylamide gels and transferred to nitrocellulose membranes via the wet tank method. Membranes were blocked for 1 hour using 5% non-fat milk, incubated overnight at 4°C with primary antibodies, washed with TBST, and incubated for 1 hour with horseradish peroxidase (HRP)-linked secondary antibodies. Protein bands were visualized using enhanced chemiluminescent detection reagents (ECL) on a LI-COR Odyssey FC. Primary antibodies were used at a 1:1000 dilution, including TWIST1 (Abcam, 50887), SPON2 (Thermo Fisher PA-106790), SPP1 (InVivoMAb BE0382), p-AKT (Ser473) (Cell Signaling, 3700S), AKT (Cell Signaling, 9272S), p44/42 MAPK (Erk1/2) (Thr202/Tyr204) (Cell Signaling, 4370S), p44/42 MAPK (Erk1/2) (Cell Signaling, 4695S), p-SRC (Tyr416) (Cell Signaling, 2101S), p-FAK (Tyr397) (Cell Signaling, 3283S), NCAD (Cell Signaling, 13116T), VIM (Cell Signaling, 5741T), SNAI2 (Cell Signaling, 9585S), ZEB1 (Cell Signaling, 3396S) and β-actin (Cell Signaling, 3700S). Secondary antibodies, anti-mouse IgG (Cell Signaling, 7076S) and anti-rabbit IgG (Cell Signaling, 7074S), were used at a 1:2000 dilution.

Immunohistochemistry analysis

Formalin-fixed, paraffin-embedded tumor tissue sections (5 μm thick) were tested using antibodies against CD4 (Cell Signaling, 25229T), CD8 (Cell Signaling, 98941T), CD11b (Cell Signaling, ab133357), SPON2 (Thermo Fisher, PA-106790), SPP1 (InVivoMAb, BE0382), CD45 (Cell Signaling, 70257S), and COL1A1 (Cell Signaling Technology, 72026S) according to standard procedures. Briefly, slides were

deparaffinized in xylene and rehydrated through a series of decreasing ethanol concentrations in water. Antigen retrieval was performed using antigen retrieval buffer (10 mM Tris-HCl, 1 mM EDTA, 10% glycerol, pH 9.0) in a pressure cooker (Biocare Medical) filled with 500 ml of water. Slides were allowed to cool to room temperature for 30 minutes followed by a PBS rinse. Primary antibodies were applied overnight in a humidified chamber at 4°C. Sections were then washed in buffer for 5 minutes, followed by a 30-minute incubation with ImmPRESS Reagent (VectorLabs, #MP7452 and #MP-7451) and then an additional two 5-minute washes. Then, sections were incubated in DAB working solution (Fisher scientific, 50-823-73) until the desired stain intensity (typically 2–5 minutes) was achieved. Finally, samples were washed twice in buffer for 5 minutes, rinsed in tap water, counterstained with hematoxylin, cleared and mounted. For immunofluorescence staining, after rinsing with PBS, slides were stained with Alexa 488-anti-mouse (Cell Signaling Technology, 4408S) and Alexa 488-anti-rabbit (Cell Signaling Technology, 8889S) secondary antibodies and mounted with Fluoromount-G with DAPI (Thermo Fisher, 00-4958-02). The slides were viewed and photographed using an ECHO Revolve Microscope. Quantification of staining was performed using ImageJ software.

Wound Healing Assay

For the wound healing assay, MDST8 or MC38 cells were plated onto 6-well tissue culture plates coated with 50 µg/ml Matrigel (BD Biosciences, San Jose, CA) with or without 100 ng/ml human SPON2 (R&D Systems, 3266-RS-025/C), 100 ng/ml mouse SPON2 (R&D Systems, 6946-RS-025), 100 ng/ml SPP1 protein (R&D Systems, 441-OP-050 or 11437-OP-050), 1 µg/ml SPON2 antibody (R&D Systems, MAB32661 or MAB3266), or 1 µg/ml

SPP1 monoclonal antibody (InVivoMAb, BE0382) with vehicle or isotype IgG controls. Once the cell monolayer reached confluency, a 200 µl pipette tip was used to create scratch wounds. The cells were washed with PBS and then cultured in medium containing 10% FBS in a tissue culture incubator. At designated time points post-scratch, plates were washed with PBS, and the wound width was photographed using an ECHO Revolve Microscope at 5x magnification and measured using ECHO Revolve Microscope software.

Haptotactic Migration and Matrigel Chemoinvasion Assays

For haptotactic migration and Matrigel chemoinvasion assays, transwells were pre-coated with 100 ng/ml recombinant mouse/human SPON2 or SPP1 protein or 1 µg/ml SPON2 or SPP1 monoclonal antibody, with or without 150 µg/cm² Matrigel. A total of 5×10^4 MDST8 or MC38 cells were added to the transwells and allowed to migrate towards medium containing 10% FBS. For the tumor cell–OmenMeso coculture system, 5×10^4 OmenMeso cells were pre-plated in the bottom chamber of the Transwell insert for 24 hours. Subsequently, a total of 5×10^4 MC38 cells were added to the upper chamber of the Transwell and allowed to migrate toward the OmenMeso cells for 48 hours. After 24 or 48 hours of incubation, cells on the top side of the transwells were removed with cotton swabs. The transwell membrane was fixed with 4% paraformaldehyde for 20 minutes, and migrated cells were stained with crystal violet and counted under a microscope at 100× magnification. Alternatively, migration and invasion quantification were conducted via colorimetric analysis. Transwell plates were destained with 7% acetic acid, and absorbance was measured at a wavelength of 590 nm using a Synergy H1 Microplate Reader.

Tumor spheroid formation assay

Spheroids were generated by culturing 120,000 cells in a 6-well ultra-low attachment plate with conditioned media (advanced DMEM/F-12) derived from L-WRN cells [24]. The media was further supplemented with 1.25 mM N-acetylcysteine (Sigma, cat # A9165), 10 mM nicotinamide (Sigma, Cat # N0636), N21-Max Media Supplement (final concentration 1X) (R&D Systems, cat # AR008), N-2MAX Media Supplement (final concentration 1X) (R&D Systems, cat # AR009), and 10 µM Y-27632 dihydrochloride (Sigma, Cat # Y-27632). When required, 100 ng/ml recombinant mouse/human SPON2 or SPP1 protein or 1 µg/ml SPON2 or SPP1 neutralization antibody with vehicle or isotype IgG controls was added to the culture medium. To propagate the spheroids into subsequent generations, they were filtered through a 40 µm cell strainer and briefly trypsinized. After a brief centrifugation, the cell pellets were resuspended in L-WRN media and replated in an ultra-low attachment plate. Spheroids were photographed using an ECHO Revolve Microscope at 5x magnification, and their diameters were measured using ECHO Revolve Microscope software.

3. **Supplemental Figures**

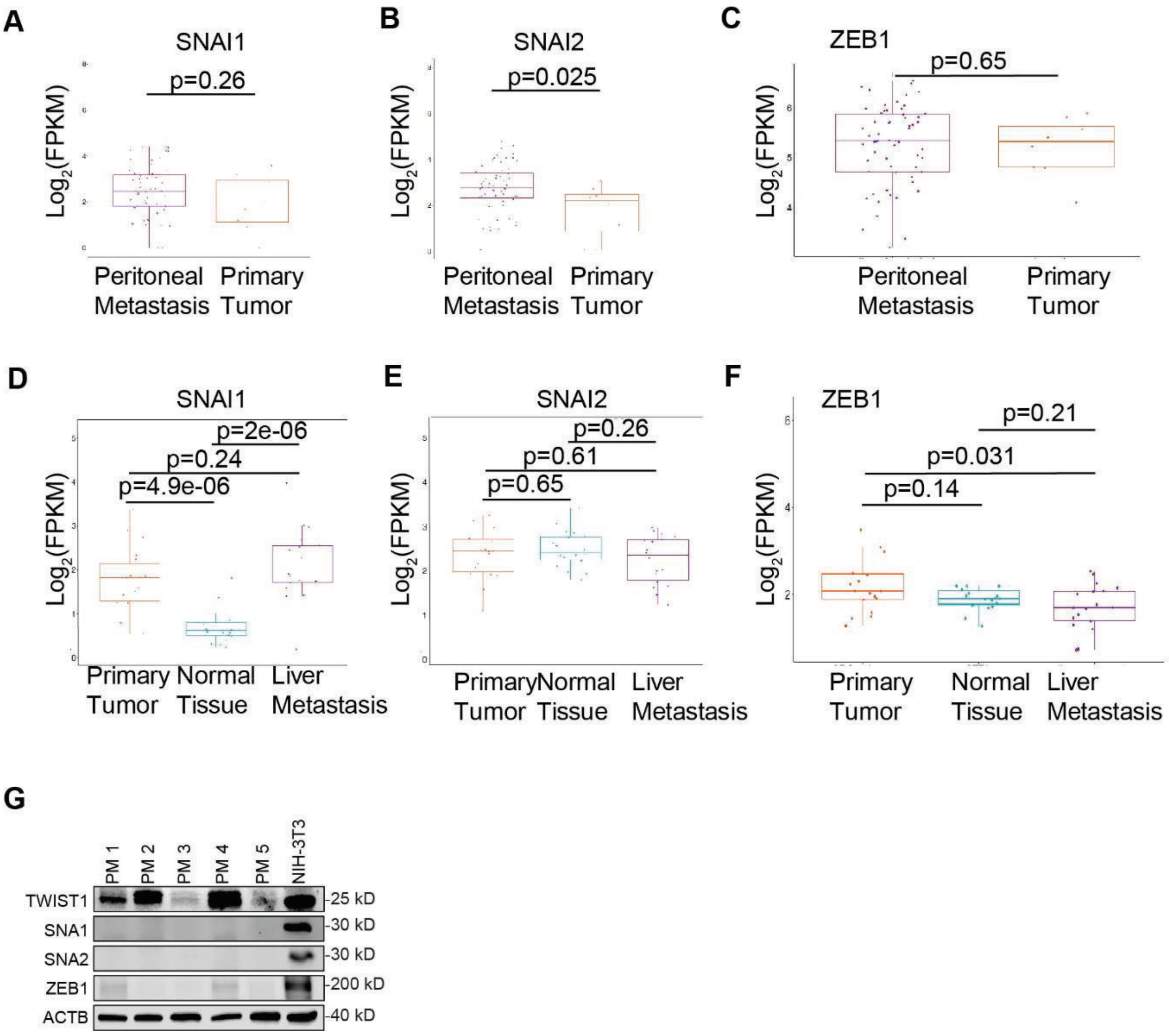

Supplemental Figure 1. Expression of EMT markers SNAI1, SNAI2, and ZEB1 in CRC peritoneal metastases (PM).
(A-C) Differential expression profiling of EMT markers (SNAI1, SNAI2, and ZEB1) in CRC peritoneal metastasis (PM) patient samples.
(D-F) Differential expression of EMT markers (SNAI1, SNAI2, and ZEB1) in CRC liver metastasis (LM) patient samples.
(G) Western blot of EMT markers (TWIST1, SNAI1, SNAI2, ZEB1) in CRC PM samples from Ohio State University Wexner Medical Center, with NIH-3T3 as positive control.

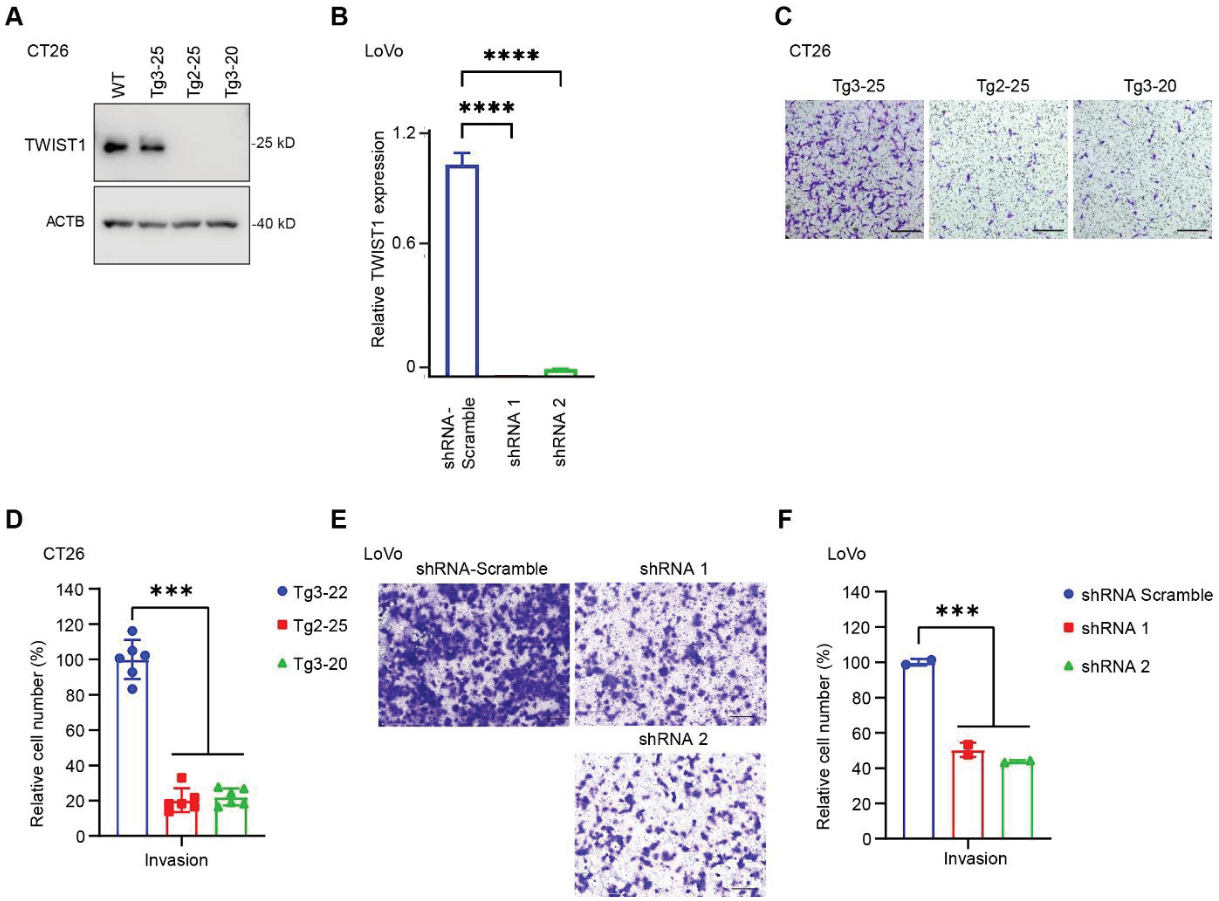

Supplemental Figure 2. TWIST1 regulates colon cancer cell migration, invasion, and stemness.

(A) Expression of TWIST1 protein in CT26 TWIST1 knockout single colony clones, confirmed by western blot.

(B) Expression of TWIST1 in LoVo cells with TWIST1 shRNA knockdown, assessed by western blot.

(C-D) Transwell migration assays of CT26 cells with TWIST1 knockout. Representative images are shown in (C), and quantification is presented in (D). Scale bar: 200 µm.

(E-F) Transwell migration assays of LoVo cells with TWIST1 knockdown. Representative images are shown in (E), and quantification is presented in (F). Scale bar: 200 µm.

Results are expressed as mean ± SD. $^{*}P < 0.05$, $^{**}P < 0.01$, $^{***}P < 0.001$, and $^{****}P < 0.0001$.

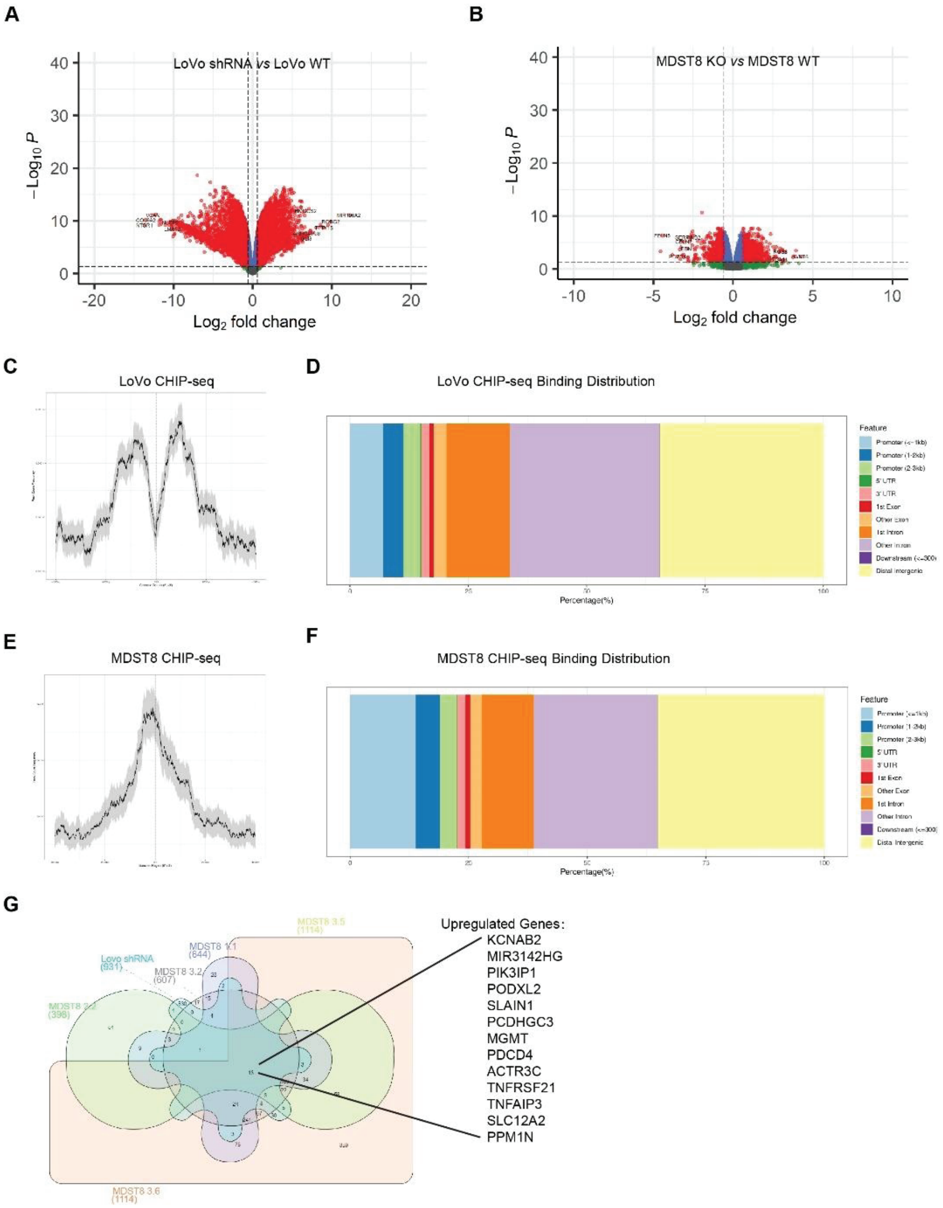

Supplemental Figure 3. RNA-seq and TWIST1 ChIP-seq analysis of LoVo and MDST8 cells.

(A) Volcano plot depicting differentially expressed genes in LoVo cells with TWIST1 knockdown (shRNA-1) versus LoVo wild-type (WT) cells.

(B) Volcano plot depicting differentially expressed genes in MDST8 TWIST1 knockout (clone 3.6) versus MDST8 WT cells.

(C) Peak count distribution of TWIST1 binding relative to the genome in LoVo cells, based on ChIP-seq analysis.

(D) Genomic feature distribution of TWIST1 binding sites in LoVo cells.

(E) Peak count distribution of TWIST1 binding relative to the genome in MDST8 cells, based on ChIP-seq analysis.

(F) Genomic feature distribution of TWIST1 binding sites in MDST8 cells.

(G) Overlay of ChIP-seq and RNA-seq data from LoVo and MDST8 cells. Venn diagrams illustrate genes with TWIST1-bound promoters (from ChIP-seq) that are upregulated in TWIST1-deficient cells, highlighting key TWIST1-regulated targets.
Results provide insight into the transcriptional and epigenetic regulation mediated by TWIST1 in colorectal cancer metastasis.

A B

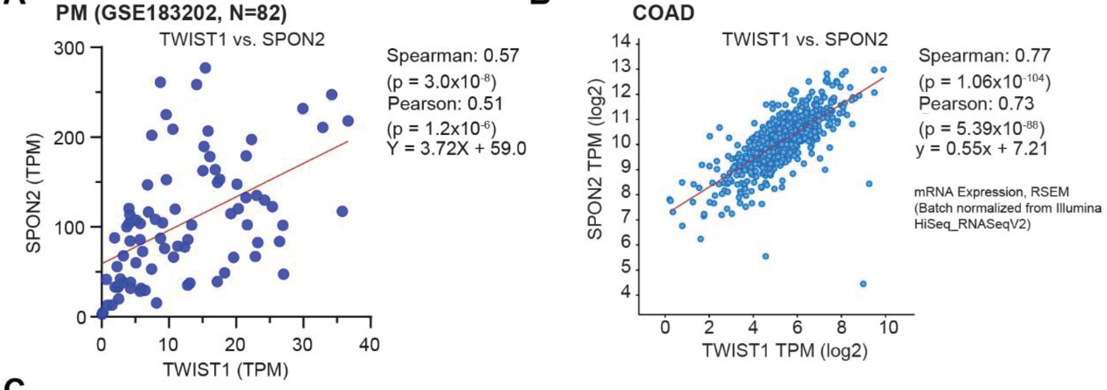

C

SPON2 in COAD (n=458)						
Stage	coef	HR	se(coef)	95%CI	z	p
1	0.004	1.004	0.093	0.837-1.206	0.047	0.962
2	0.754	2.125	0.54	0.737-6.124	1.396	0.163
3	1.312	3.712	0.537	1.296-10.636	2.443	0.015
4	2.369	10.692	0.535	3.746-30.512	4.429	0.000

R^2=0.1
Likelihood ratio test p=4.29e-0.9
Wald test p=1.63e-09
Score (logrank) test p=7.16e-12

Supplemental Figure 4. SPON2 Expression as a Poor Prognostic Marker in PM Patient Samples and Its Correlation with TWIST1 Expression
(A) Significant positive correlation between TWIST1 and SPON2 expression in CRC peritoneal metastasis (PM) patient samples (GEO: GSE183202), indicating a potential regulatory relationship.
(B) TCGA data analysis showing a significant positive correlation between TWIST1 and SPON2 expression in CRC patients, further supporting the association observed in PM samples.
(C) Increasing hazard ratio for TCGA colon cancer samples, stratified by stage, demonstrating that higher SPON2 expression is associated with worse prognosis across disease stages.

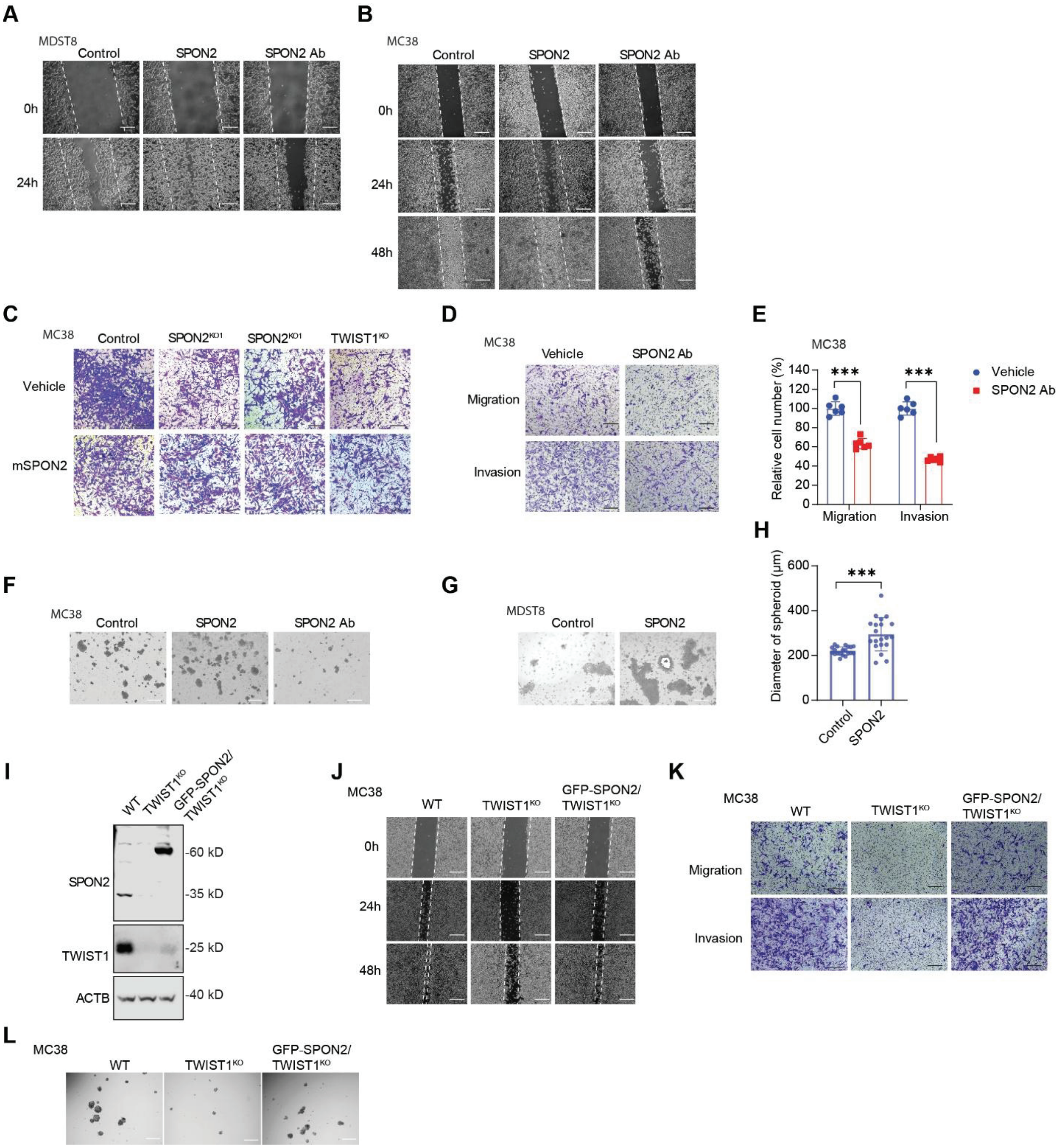

Supplemental Figure 5. The TWIST1-SPON2 Cascade Regulates Colon Cancer Cell Migration, Invasion, and Stemness

(A-B) Wound-healing assay to assess the migration of MC38 (A) and MDST8 (B) cells, plated on Matrigel-coated plates with or without 100 ng/ml SPON2 protein or 1 µg/ml SPON2 monoclonal antibody. Representative images shown. Scale bar, 200 µm.

(C) Transwell Matrigel invasion assays to evaluate the effect of SPON2 protein on the chemoinvasion of MC38 TWIST1 or SPON2 knockout cells. The Transwell was coated with 1 mg/ml Matrigel with or without 100 ng/ml SPON2 protein and incubated for 48 hours with 10% FBS. Representative images shown. Scale bar, 200 µm.

(D-E) Transwell migration and Matrigel invasion assays to assess haptotactic migration and Matrigel chemoinvasion in MC38 cells with or without 100 ng/ml SPON2 protein. Representative images are shown in (D), and statistical results are presented in (E). Scale bar, 200 μm.
(F) Assessment of self-renewal capacity in MC38 cells cultured with 100 ng/ml SPON2 protein or 1 μg/ml SPON2 monoclonal antibody.
(G-H) Assessment of self-renewal capacity in MDST8 cells cultured with 100 ng/ml SPON2 protein. Representative images shown in (G) and statistical results shown in (H). Scale bar, 200 μm.
(I) Western blot showing the expression of GFP-SPON2 in MC38 cells with TWIST1 knockout.
(J) Wound-healing assay to assess the migration of MC38 TWIST1 knockout cells with overexpression of GFP-SPON2. MC38 cells were plated on Matrigel-coated plates.
(K) Transwell migration and Matrigel invasion assays to assess haptotactic migration and Matrigel chemoinvasion of MC38 TWIST1 knockout cells with overexpression of GFP-SPON2.
(L) Self-renewal capacity of MC38 TWIST1 knockout cells with overexpression of GFP-SPON2.
Results are expressed as mean ± SD. *$P < 0.05$, **$P < 0.01$, ***$P < 0.001$, and ****$P < 0.0001$.

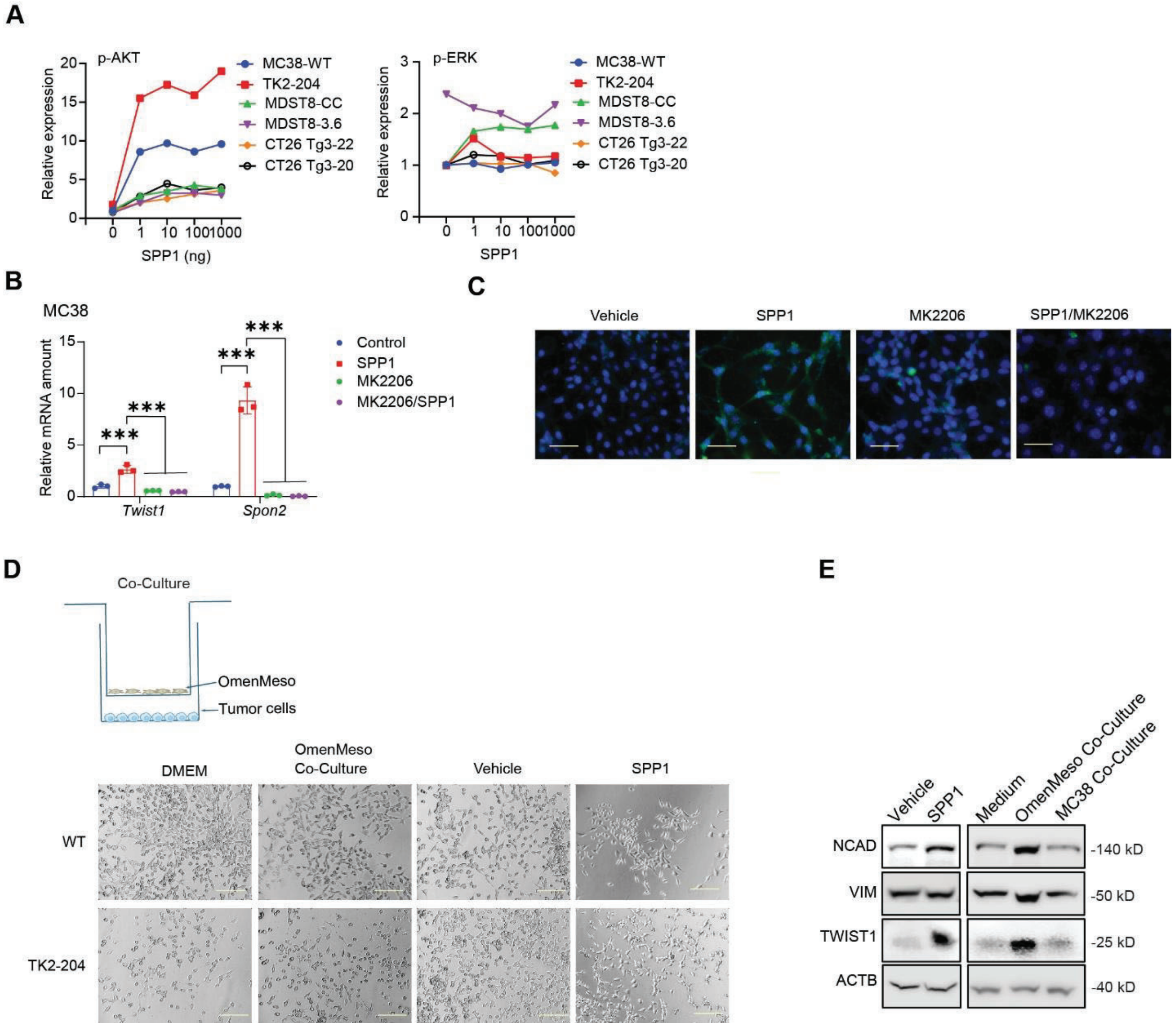

Supplemental Figure 6. The SPP1 Enhances TWIST1-SPON2 Cascades and EMT

(A) Quantification of relative p-AKT and p-ERK protein expression in MC38, MDST8 and CT26 cells from the western blot of Figure 4E.

(B) Expression of *Twist1* and *Spon2* mRNA in MC38 cells treated with 100 ng/ml SPP1 protein with or without 1 μM MK2206 for 24 hours.

(C) Expression of *Spon2* promoter-GFP in MC38 cells treated with 100 ng/ml SPP1 protein and 1 μM AKT inhibitor MK2206 for 24 hours.

(D) Top: Schematic illustration of the transwell co-culture system involving Omental Mesothelial cells (OmenMeso) and tumor cells. Bottom: Phase-contrast microscopy images showing the morphology of Wild Type (WT) and TK2-204 cells cultured in control medium (DMEM), OmenMeso co-culture, Vehicle, or SPP1-containing medium.

(E) Western blot analysis of EMT markers (NCAD, VIM, TWIST1) in cells treated with SPP1 (left) or co-cultured with OmenMeso cells (right). ACTB served as the loading control.

Results are expressed as mean ± SD. Statistical significance was determined with ***P < 0.001.

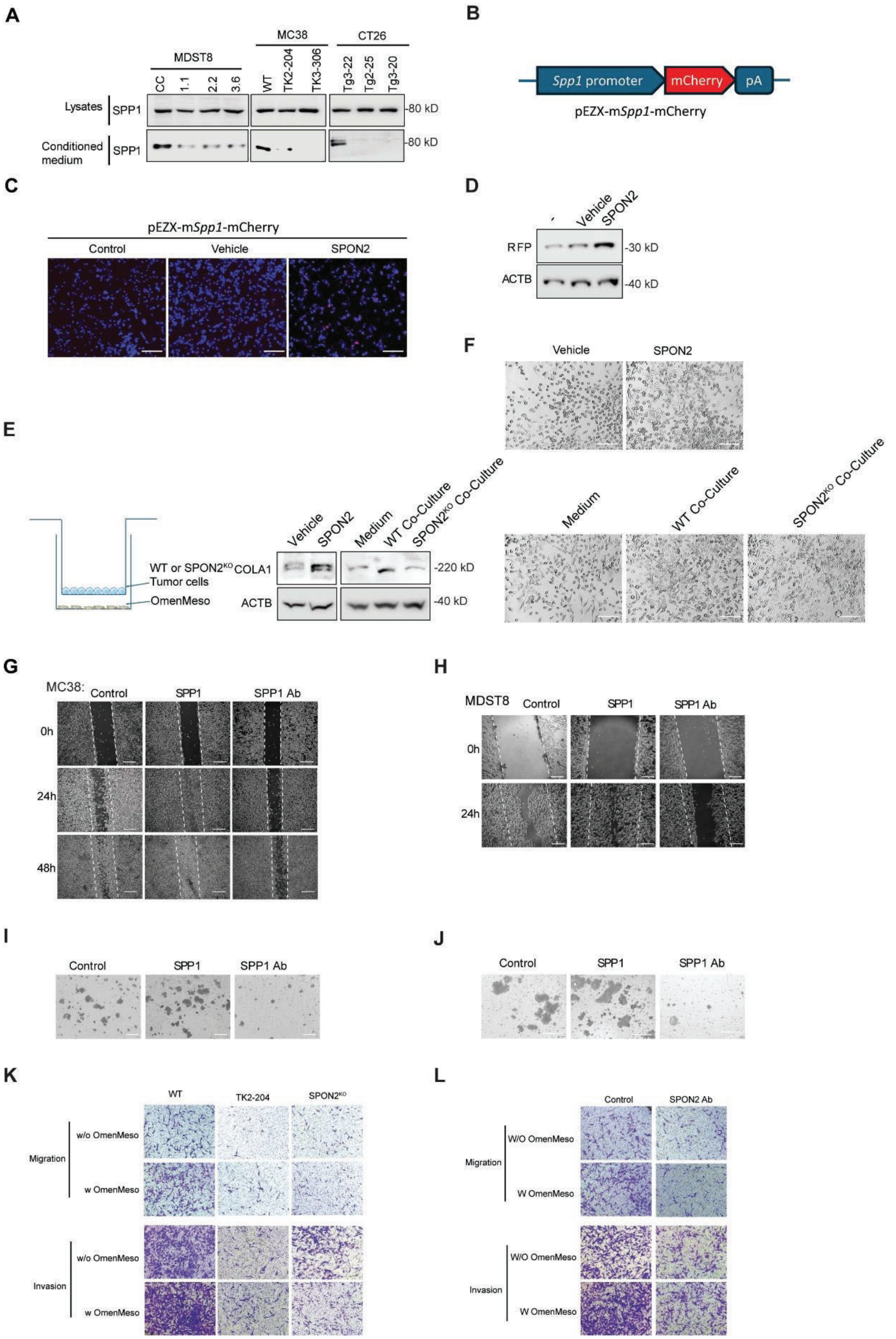

Supplemental Figure 7. The SPON2 increases SPP1 Expression in Mesothelial Cells and Enhances Colon Cancer Cell Migration, Invasion, and Stemness

(A) Western blot analysis of SPP1 expression in whole-cell lysates and conditioned medium from TWIST1 knockout MDST8, MC38, and CT26 cells.
(B) The schematic figure shows the structure of p-EZX-m*Spp1*-mCherry.
(C-D) Expression of *Spp1* promoter-mCherry in OmenMeso cells treated with 100 ng/ml SPON2 protein for 24 hours. Representative images are shown in (C), and Western blotting results are shown in (D).
(E) Western blot analysis of cancer-associated fibroblast (CAF) markers COL1A1 expression in WT or SPON2 knockout ($SPON2^{KO}$) tumor cells co-cultured with OmenMeso cells or treated with vehicle or 100 ng/ml SPON2 protein for 24h. ACTB serves as loading control. Schematic of the co-culture system is shown on the left.
(F) Representative brightfield images of OmenMeso cells grown in medium alone, in co-culture with wild-type or SPON2 knockout MC38 cells. Scale bars are indicated.
(G-H) Wound-healing assays to evaluate the migration of MC38 (G) and MDST8 (H) cells. MC38 and MDST8 cells were plated on Matrigel-coated plates with or without 100 ng/ml SPP1 protein or 1 µg/ml SPP1 monoclonal antibody.
(I-J) Self-renewal capacity of MC38 (I) and MDST8 (J) cells cultured with 100 ng/ml SPP1 protein or 1 µg/ml SPP1 monoclonal antibody.
(K–L) Transwell migration and invasion assays of tumor cells co-culture with OmenMeso cells. (K) Migration (upper chamber) and invasion (lower chamber) of wild-type (WT), TK2-204, or $SPON2^{KO}$ cells in the presence (w OmenMeso) or absence (w/o OmenMeso) of OmenMeso cells. (L) Migration and invasion of cells treated with vehicle (Control) or SPON2 neutralizing antibody (SPON2 Ab) in the presence or absence of OmenMeso cells. Cells were stained with crystal violet; representative images are shown.

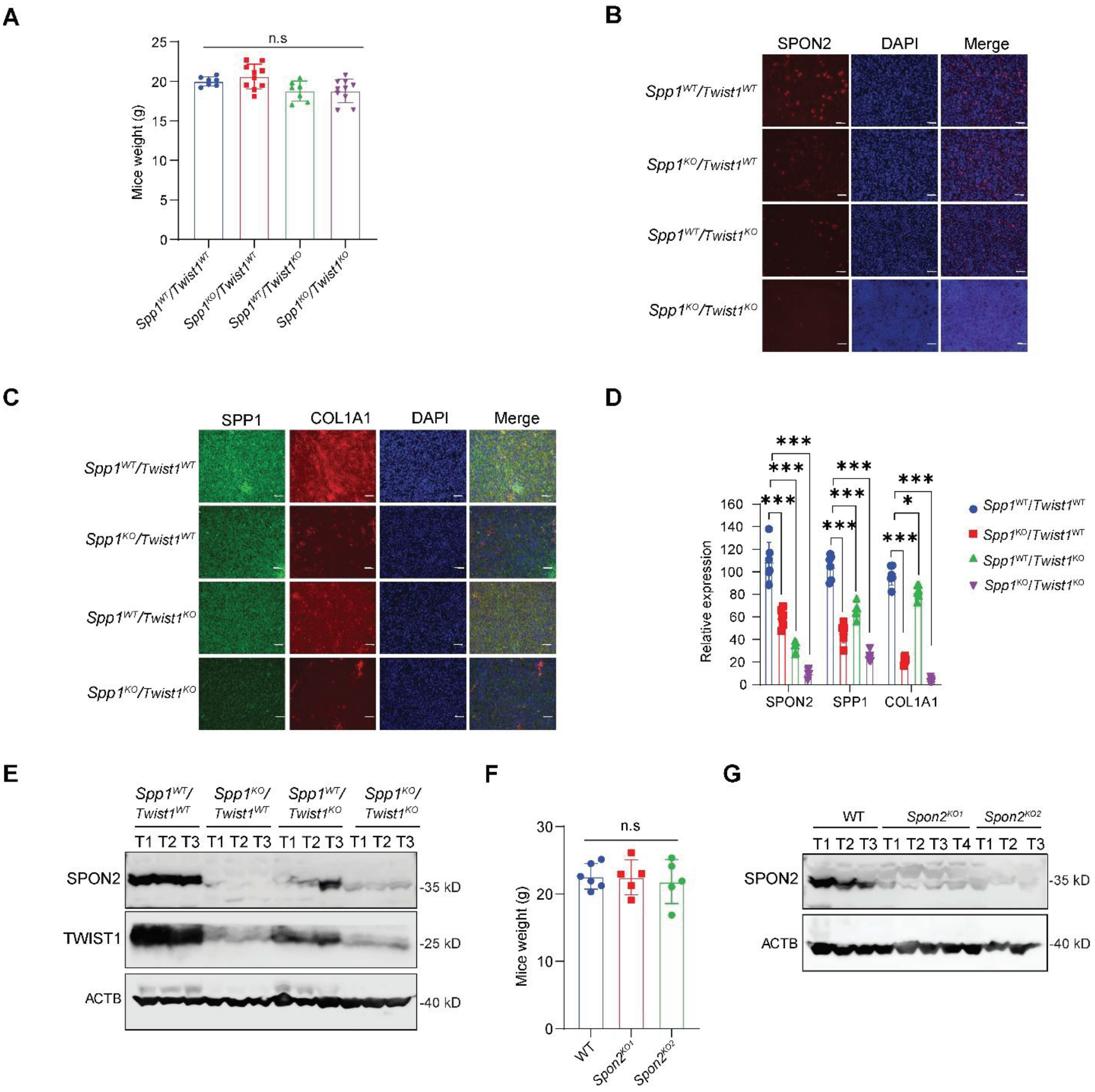

Supplemental Figure 8. The TWIST1-SPON2-SPP1 Cascades Regulate Peritoneal Metastasis

(A) Mouse weight 28 days after tumor cell injection. C57BL/6J mice, with or without *Spp1* gene knockout, were injected with 5 x 10^4 MC38 cells, with or without *Twist1* knockout.

(B) Immunofluorescence staining of SPON2 expression in tissue sections of MC38 peritoneal metastases.

(C) Immunofluorescence staining of SPP1 and COL1A1 expression in tissue sections of MC38 peritoneal metastases. Scale bar, 50 μm.

(D) Quantification of SPON2, SPP1, and COL1A1 expression was performed using immunofluorescence staining. Data was collected from six independent images per sample group.

(E) Western blotting of SPON2 and TWIST1 expression in MC38 peritoneal metastases in C57BL/6J mice, with or without *Spp1* gene knockout.

(F) Mouse weight 28 days after MC38 cells with or without *Spp1* gene knockout injection in C57BL/6J mice.

(G) Western blotting of SPON2 and TWIST1 expression in MC38 peritoneal metastases.

4. Uncropped gels

Figure 1

Figure 1B

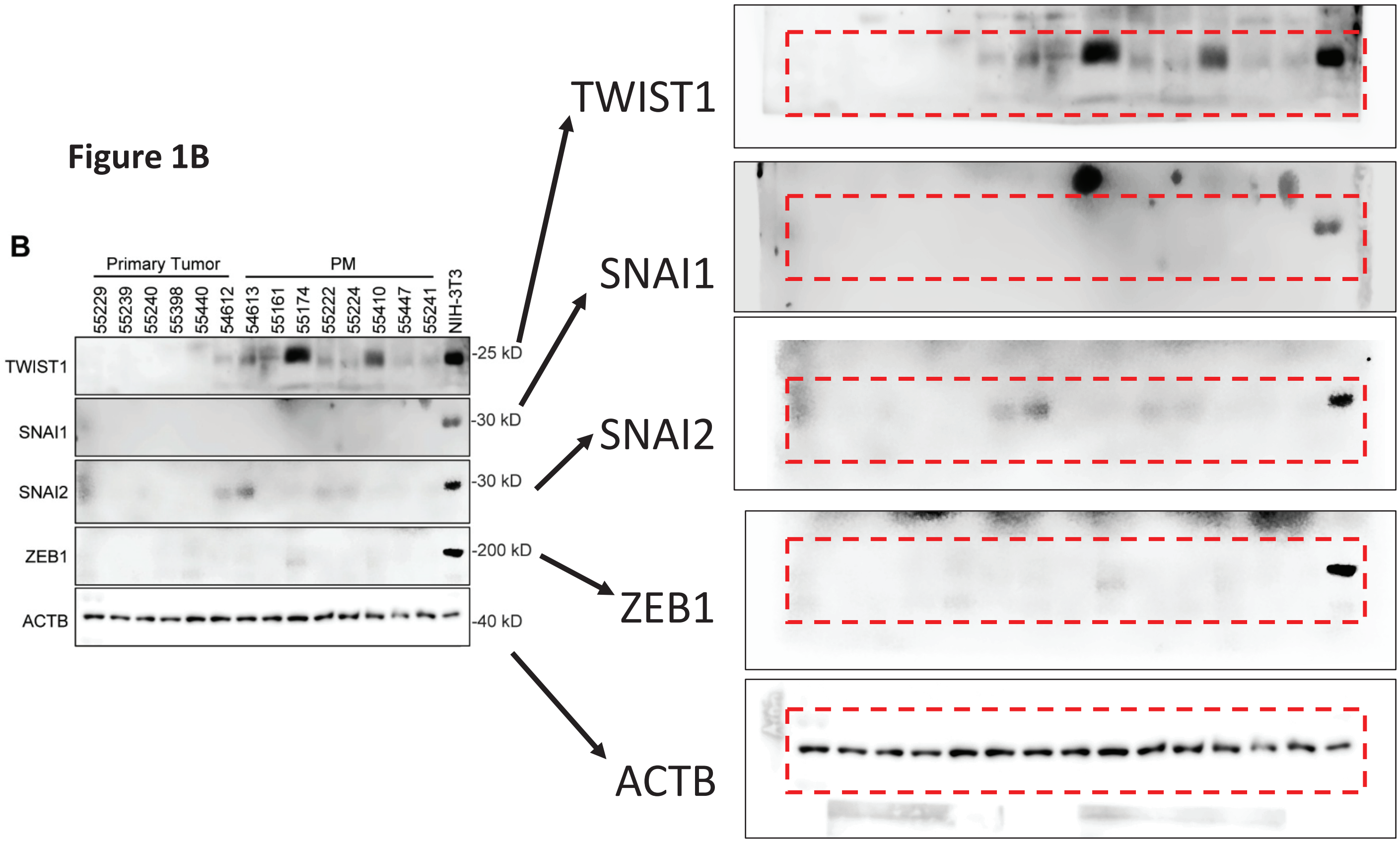

Figure 1D

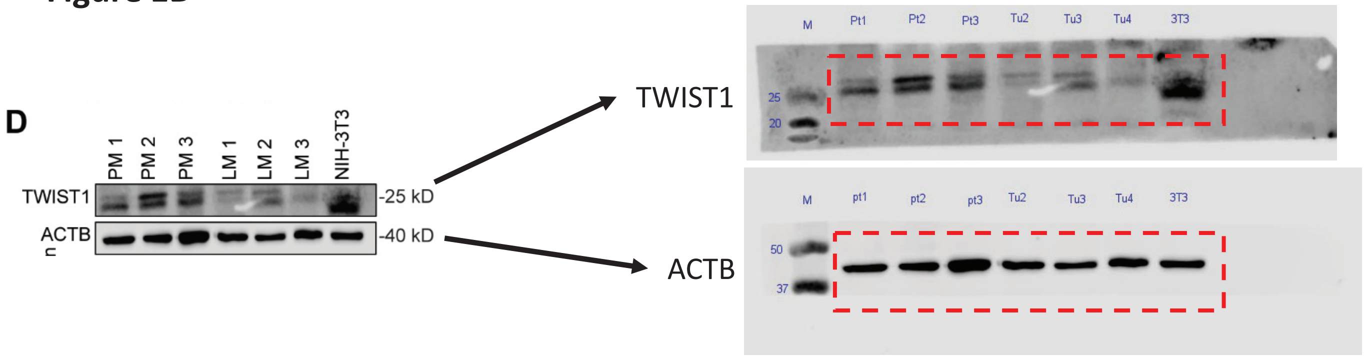

Figure 1

Figure 1E

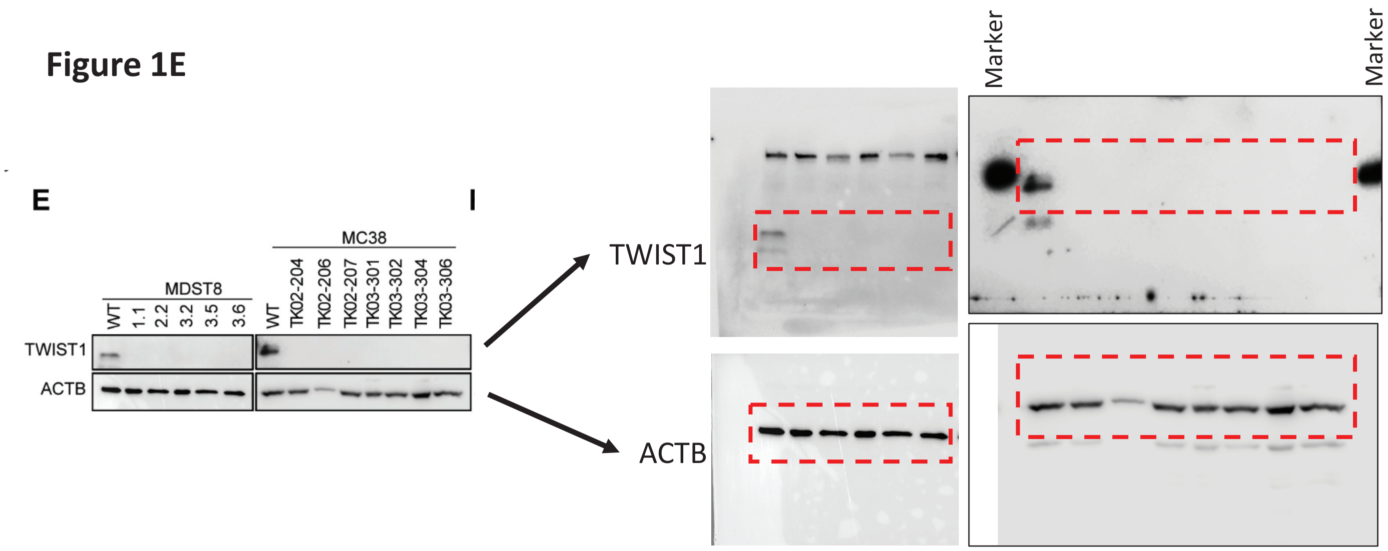

Figure 2

Figure 2A

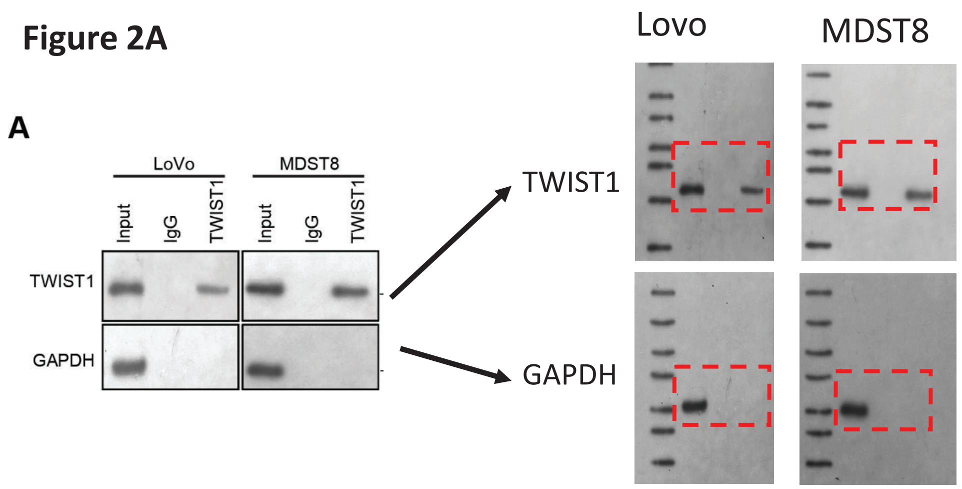

Figure 2J

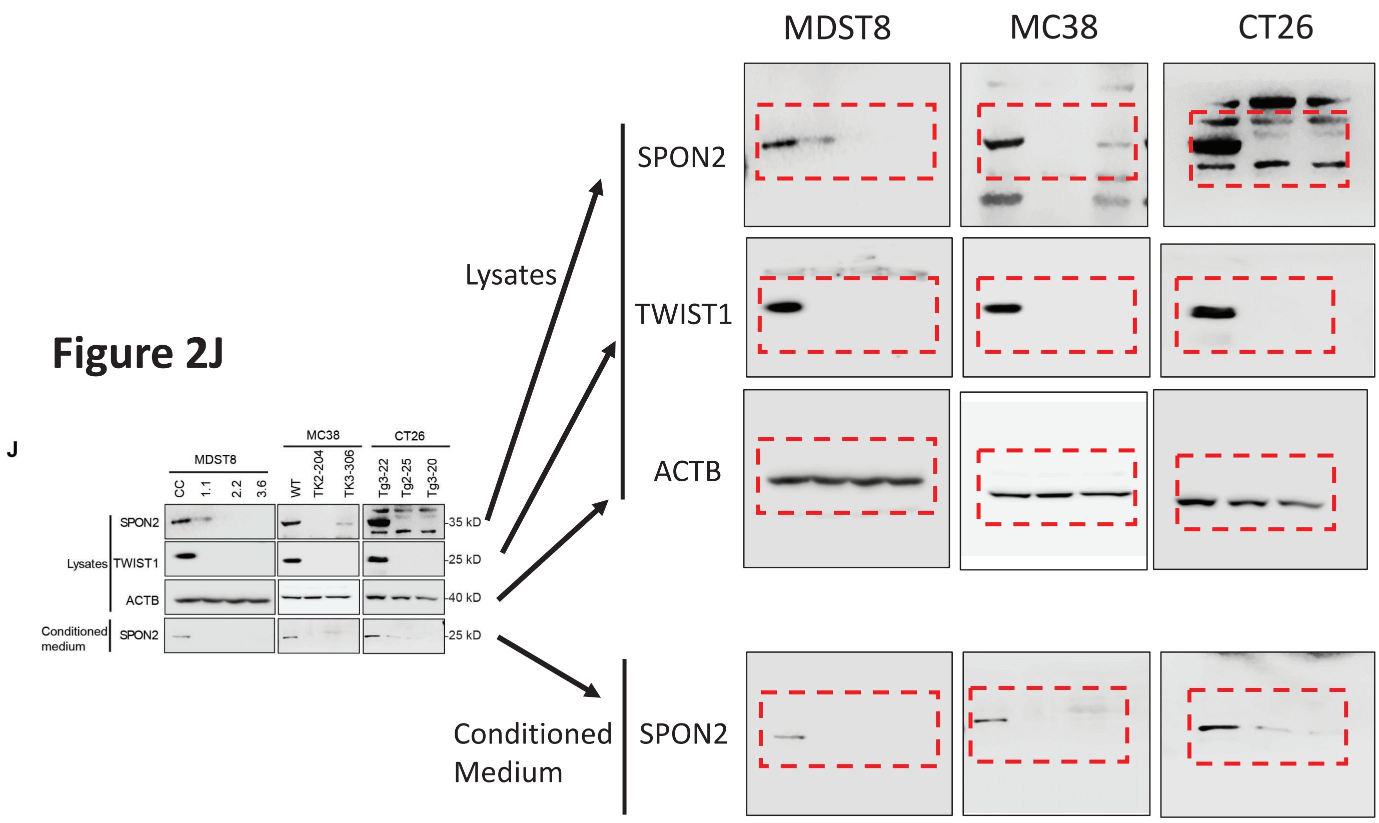

Figure 2

Figure 2M

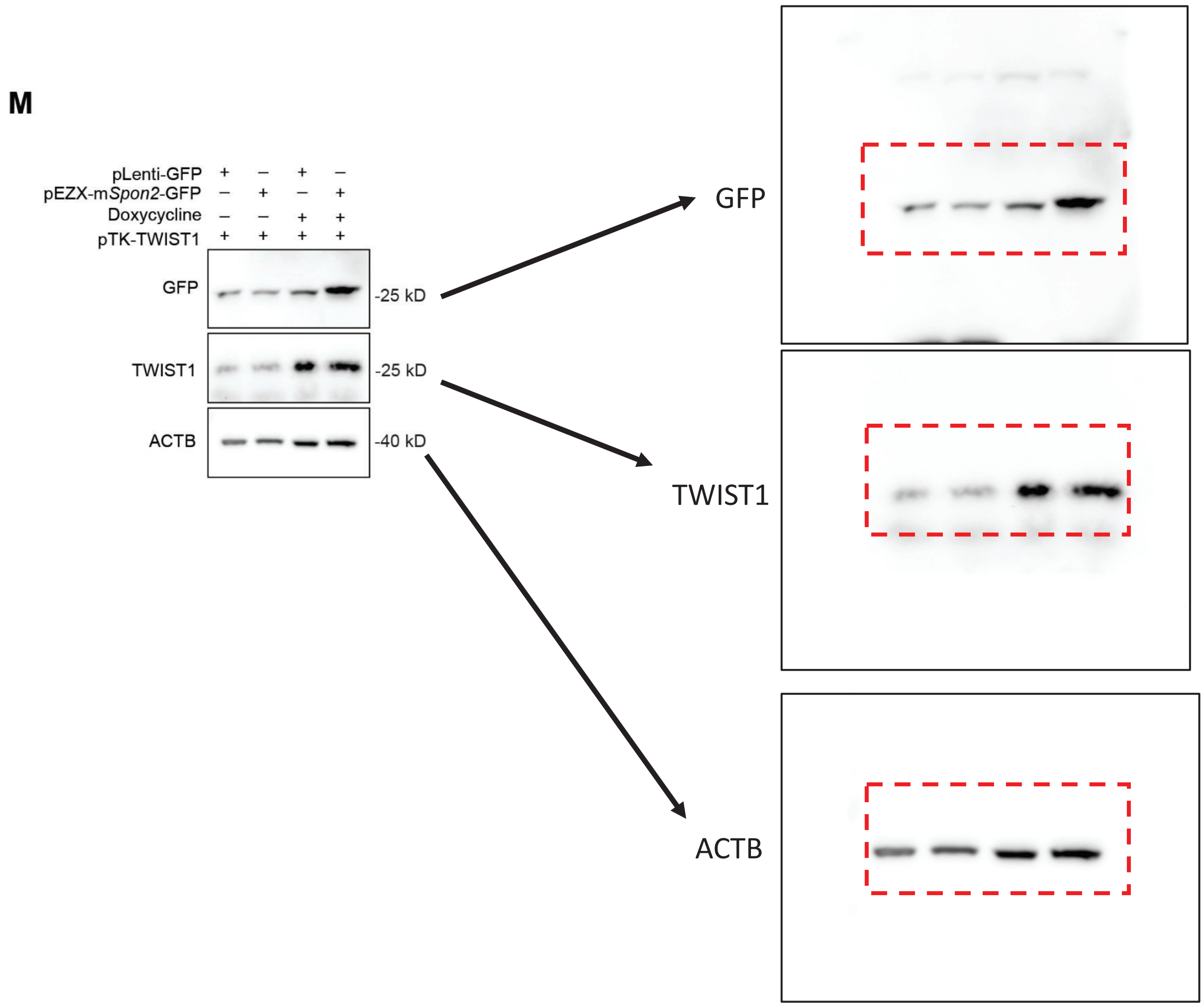

Figure 3

Figure 3A

A

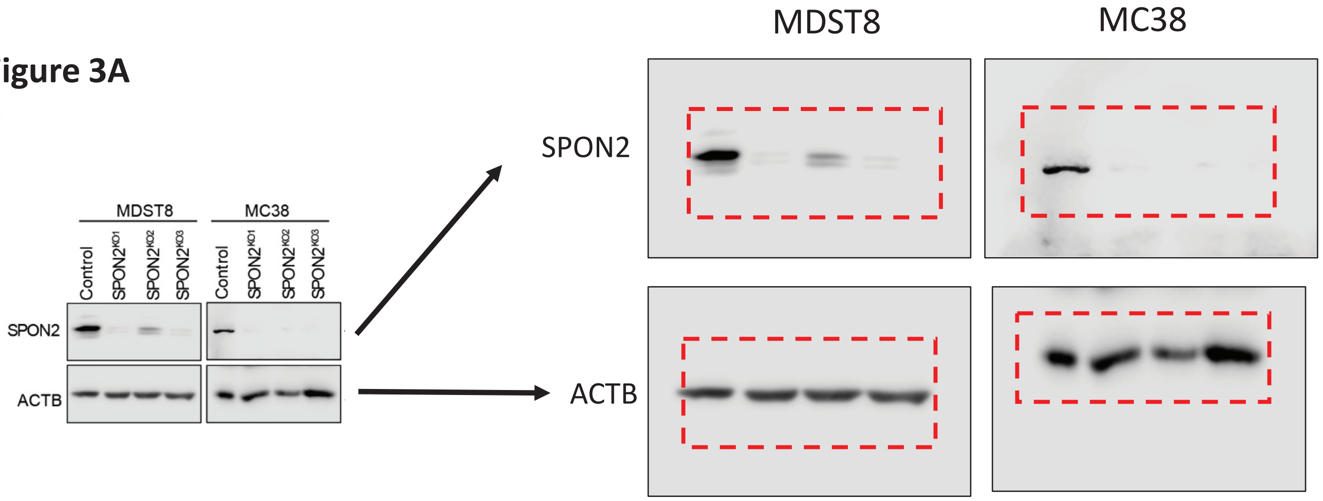

Figure 4

Figure 4A

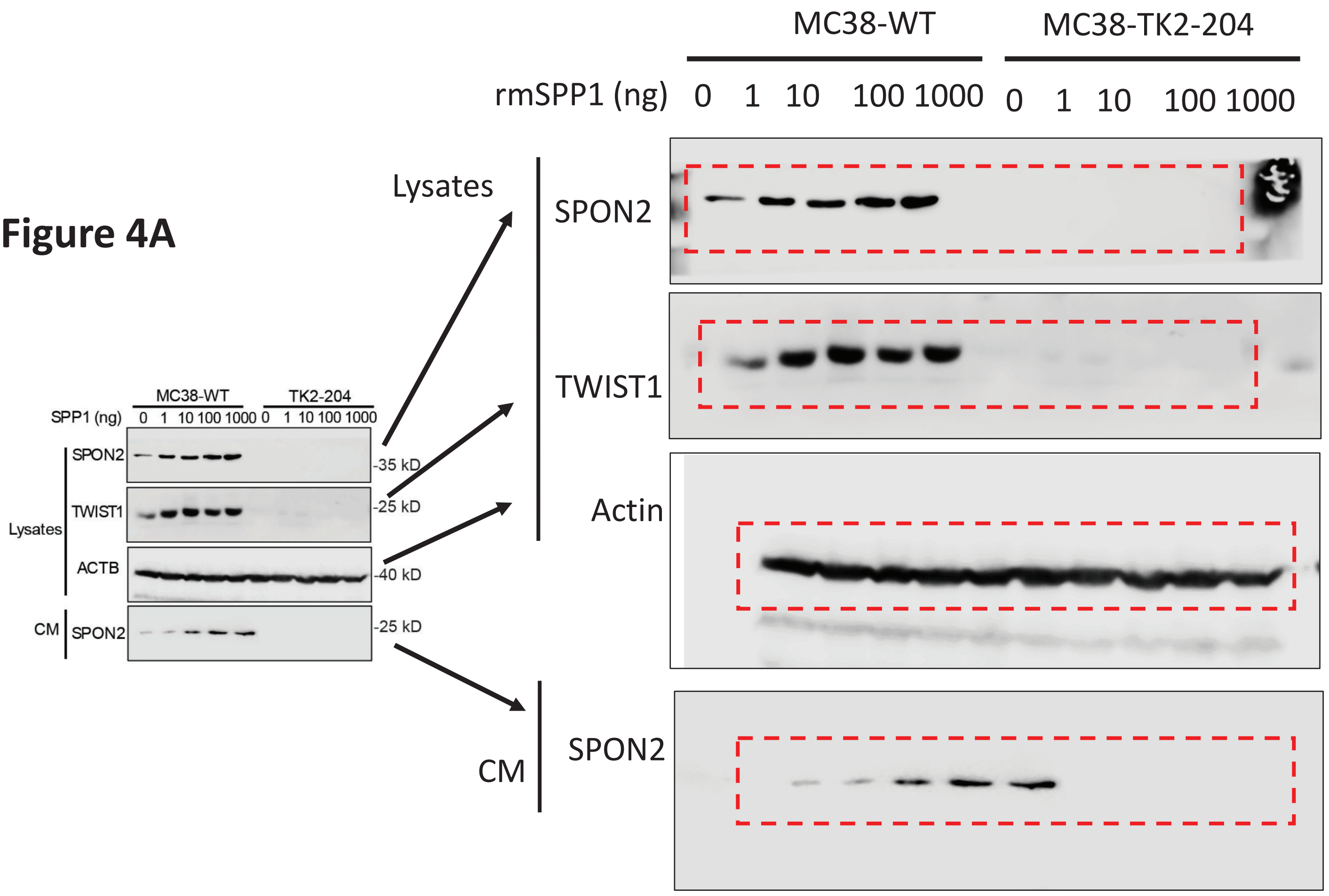

Figure 4D

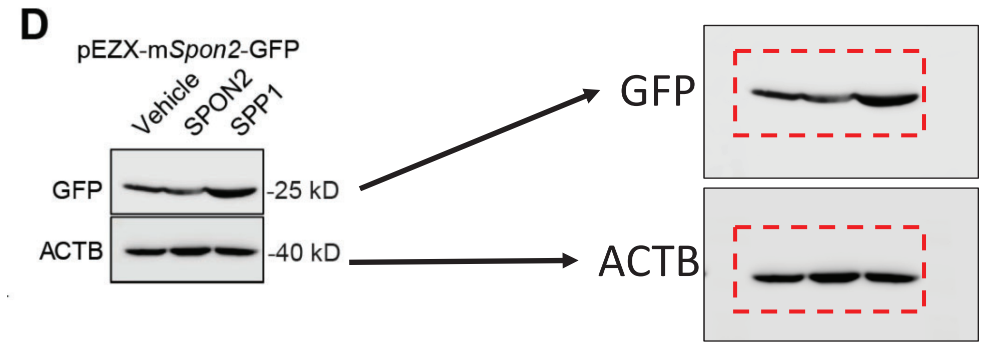

Figure 4

Figure 4E

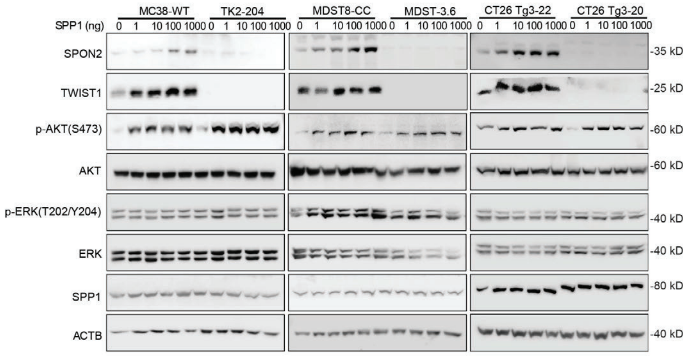

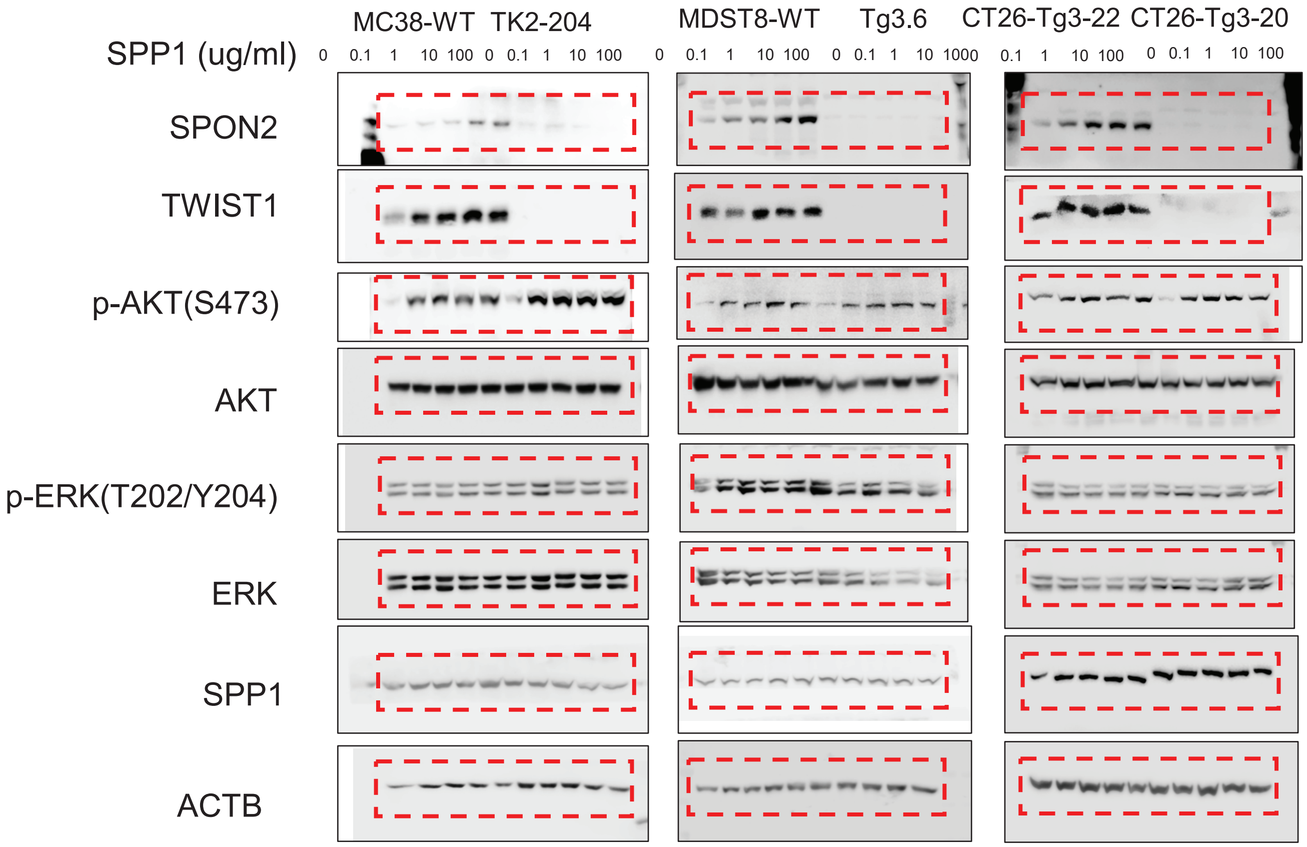

Figure 4

Figure 4F

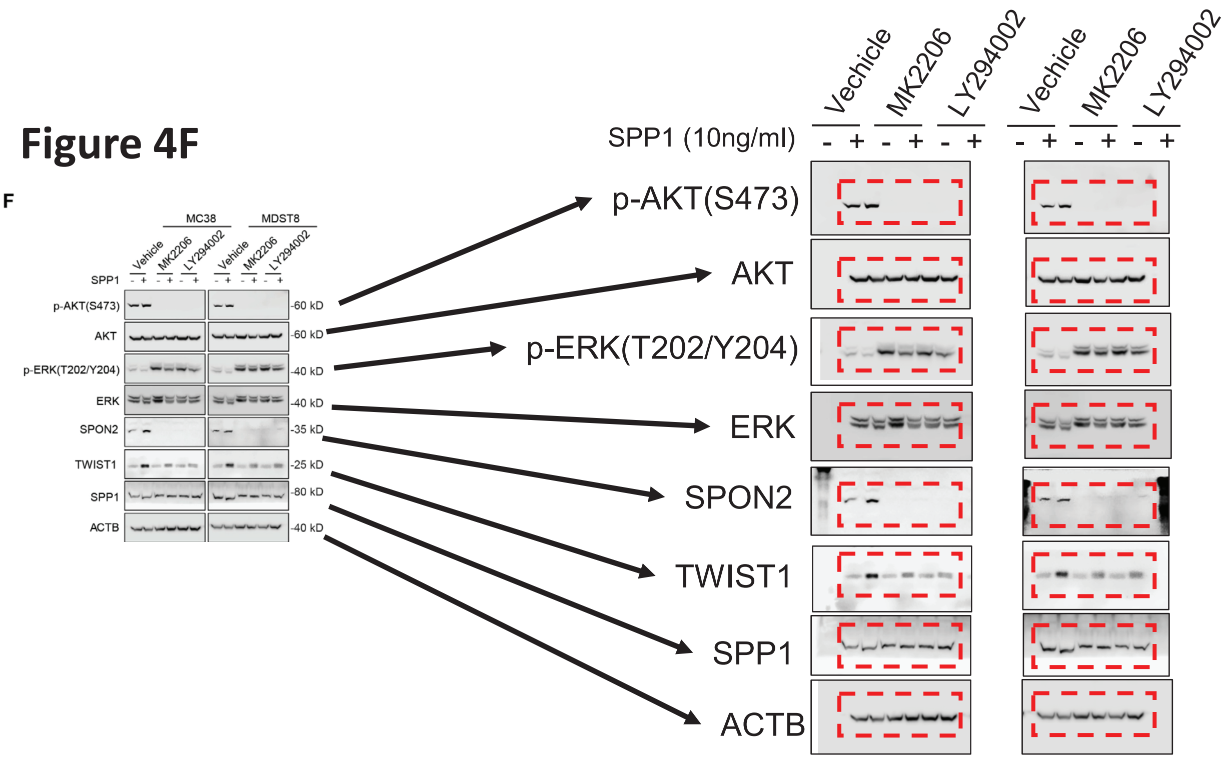

Figure 4

Figure 5G

Figure 4G

G

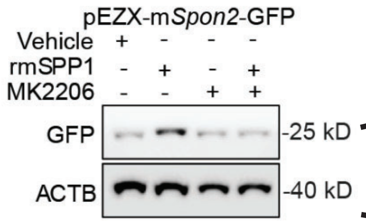

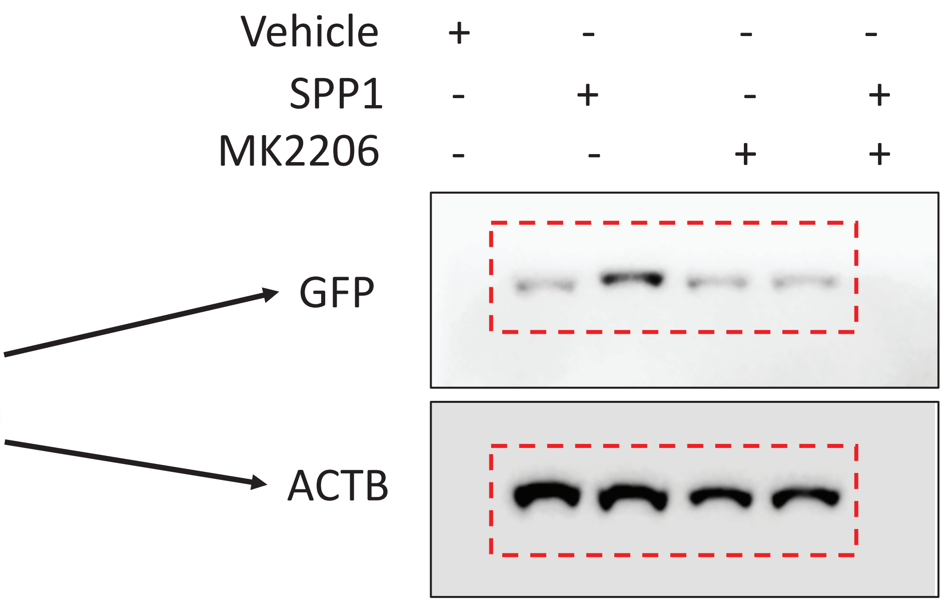

Figure 5H

Figure 4H

H

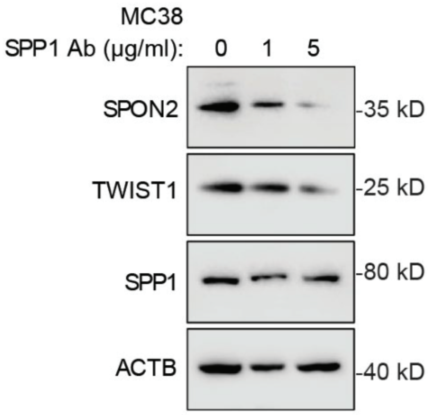

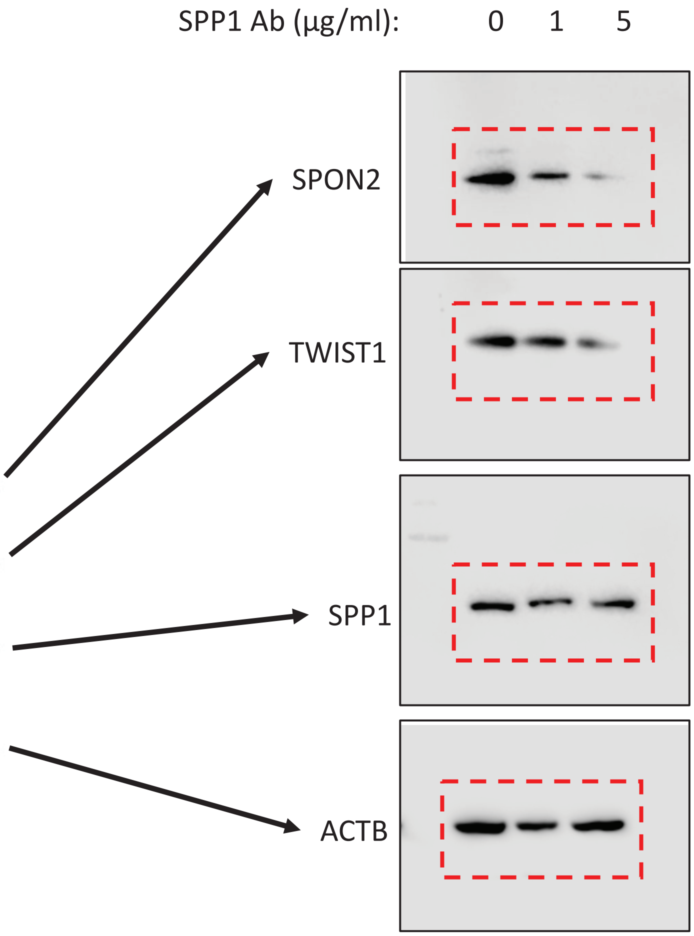

Figure 5

Figure 5A

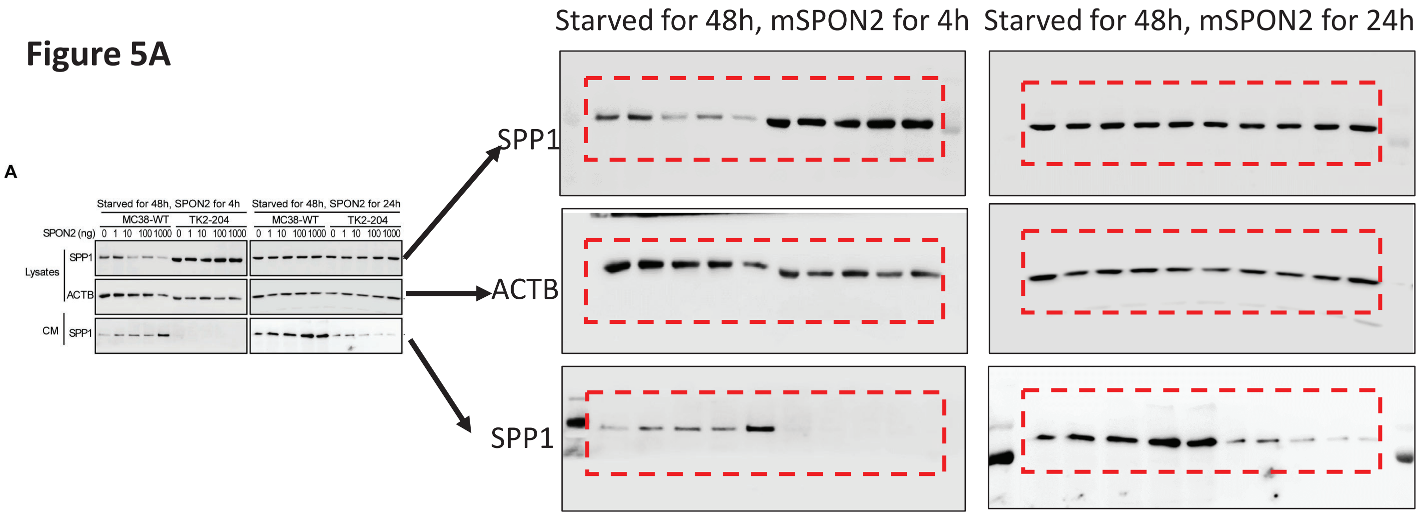

Figure 5B

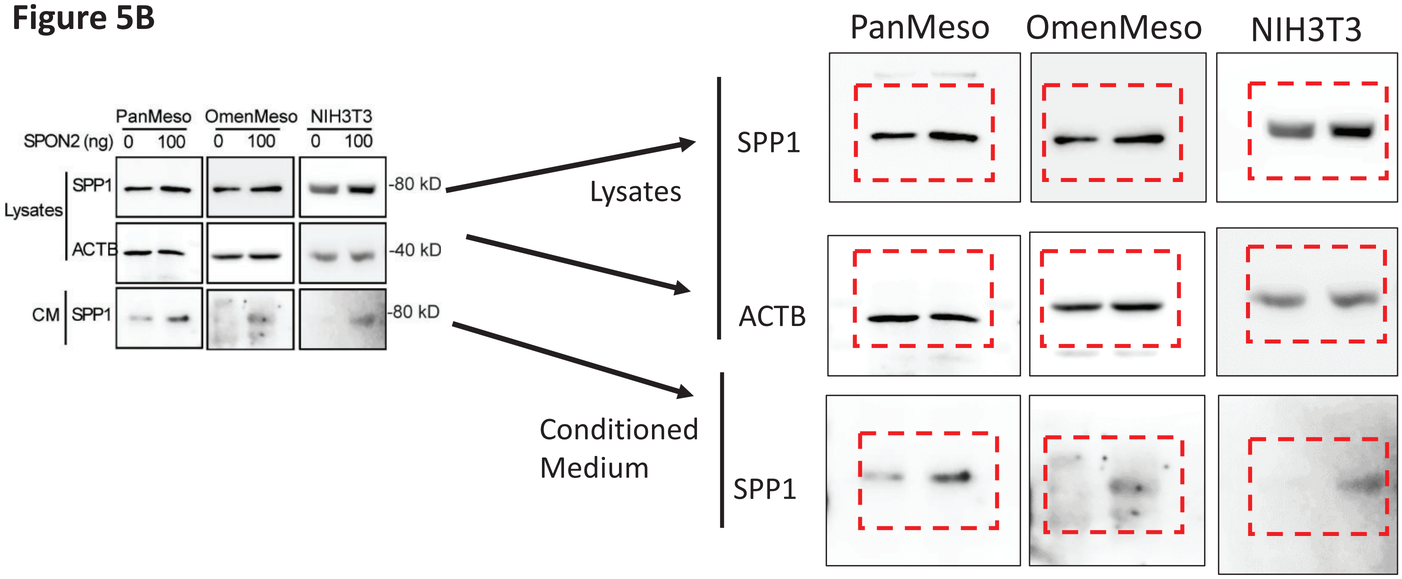

Figure 5C

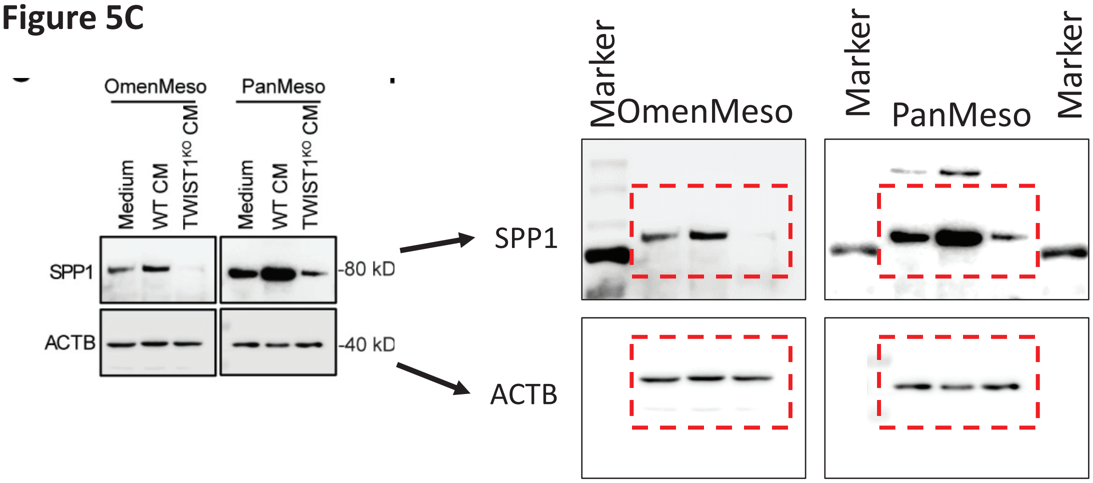

Figure 5

Figure 5D

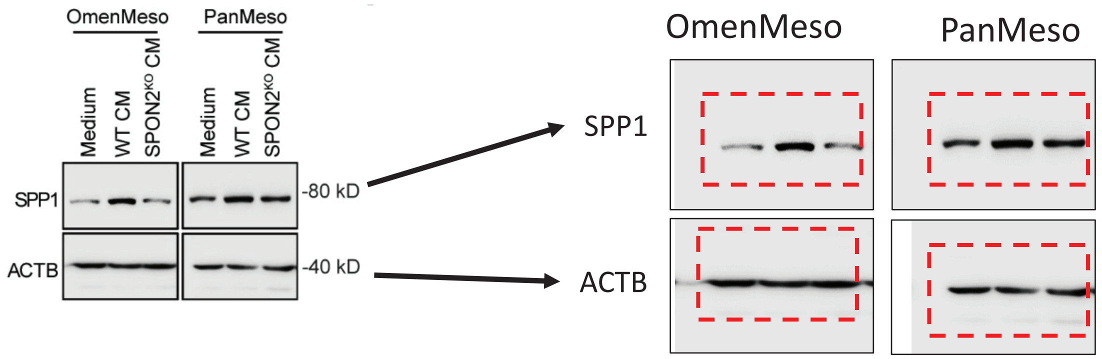

Figure 5G

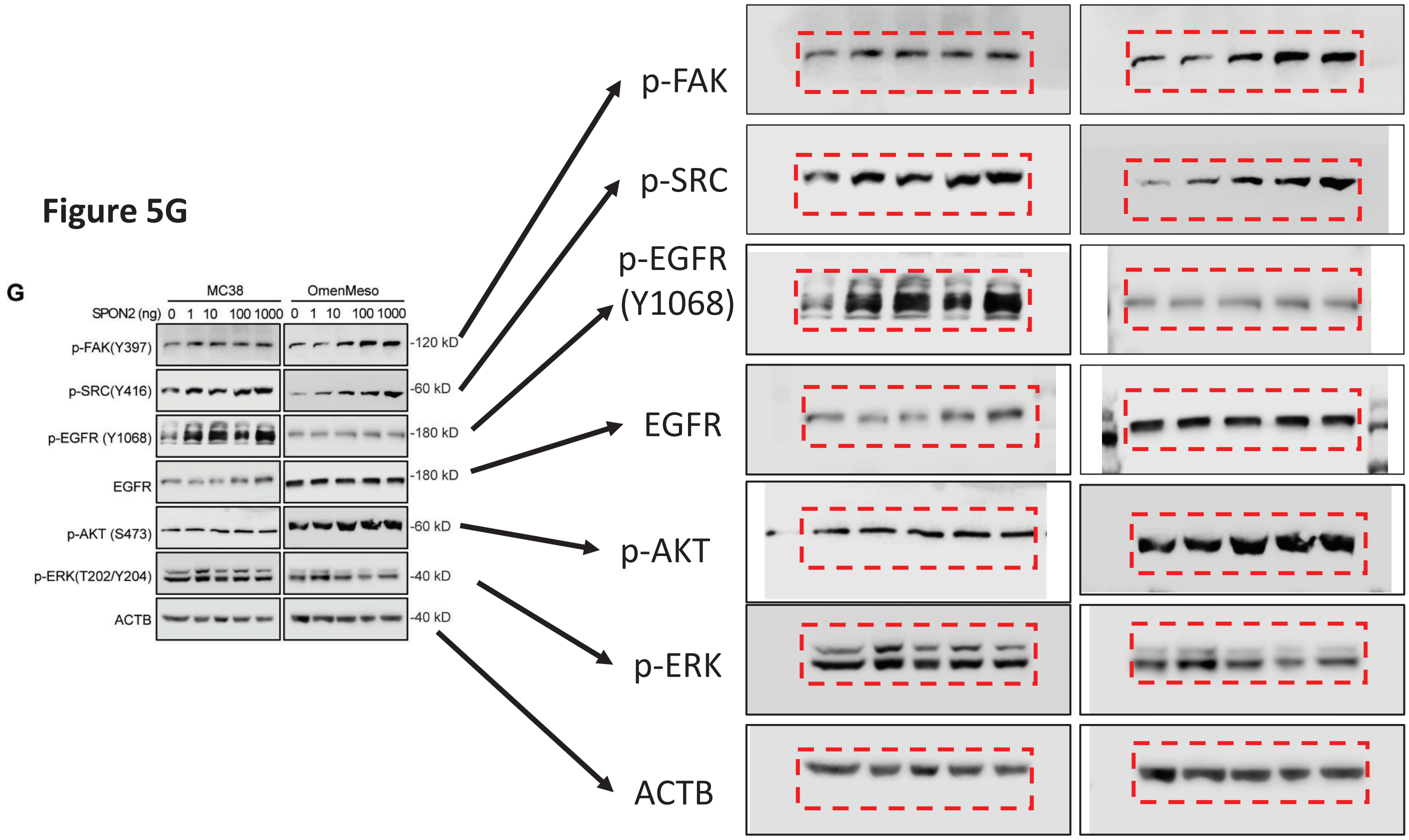

Figure 5

Figure 5H

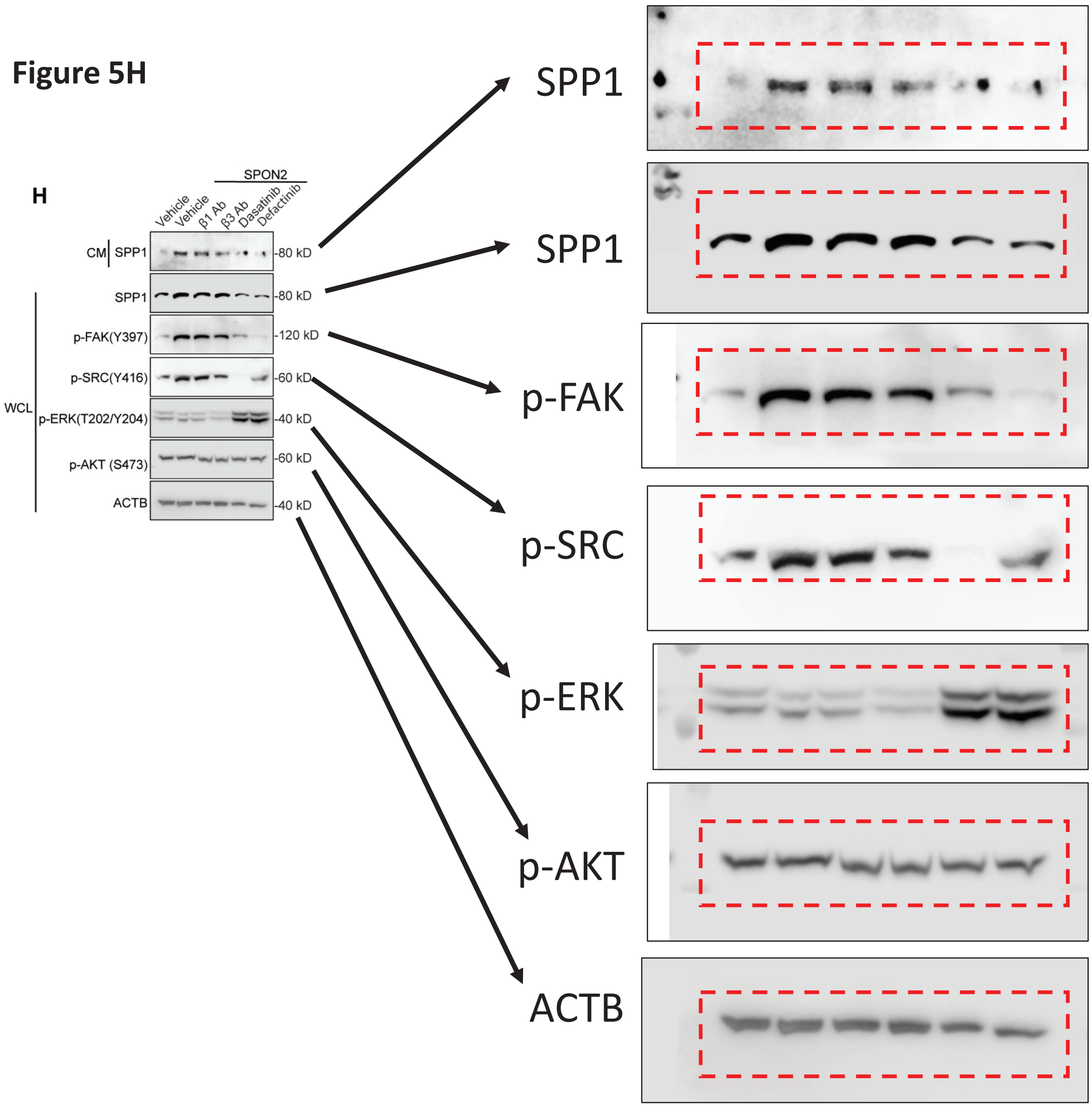

Supplemental Figure 1

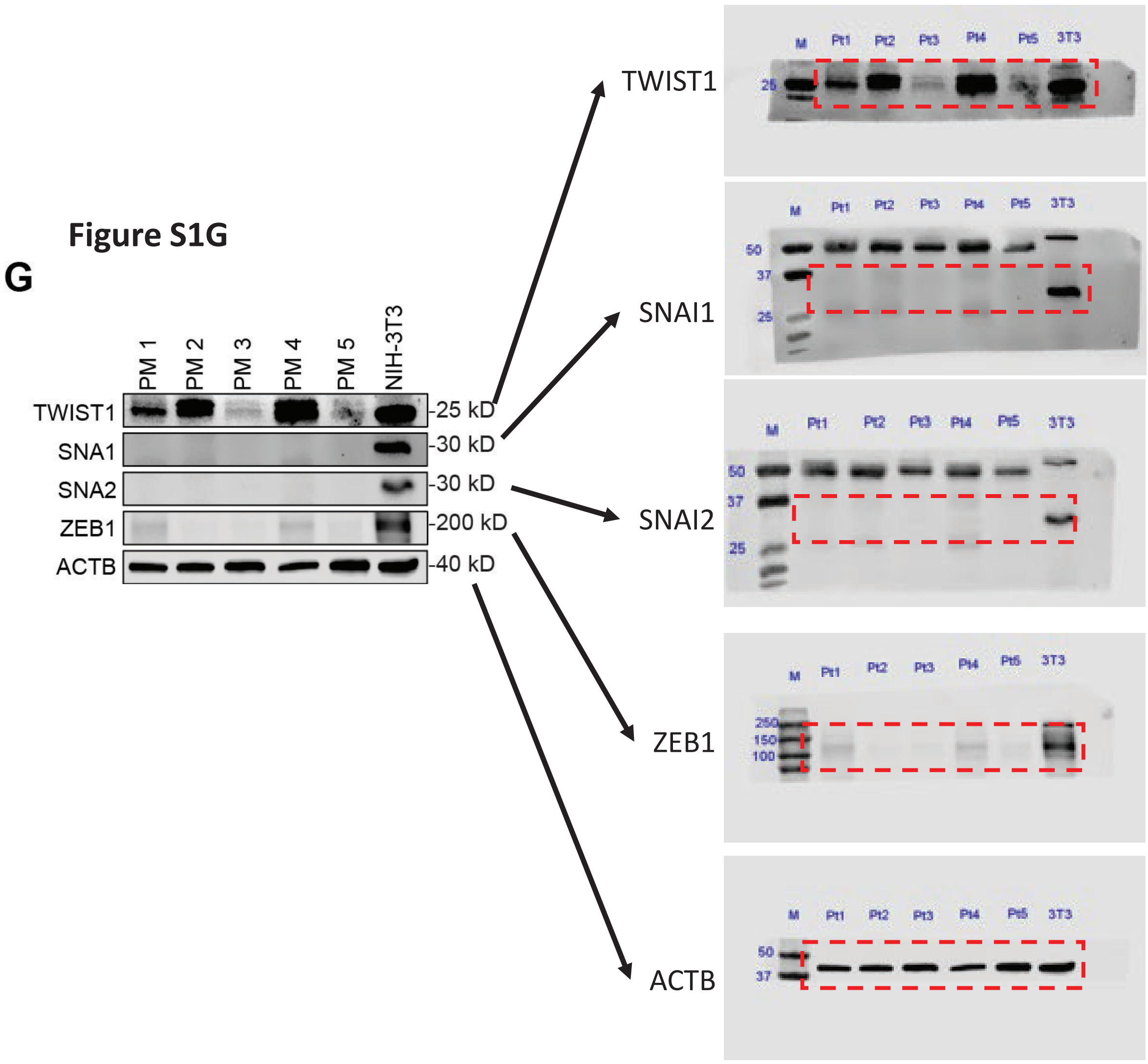

Supplemental Figure 2

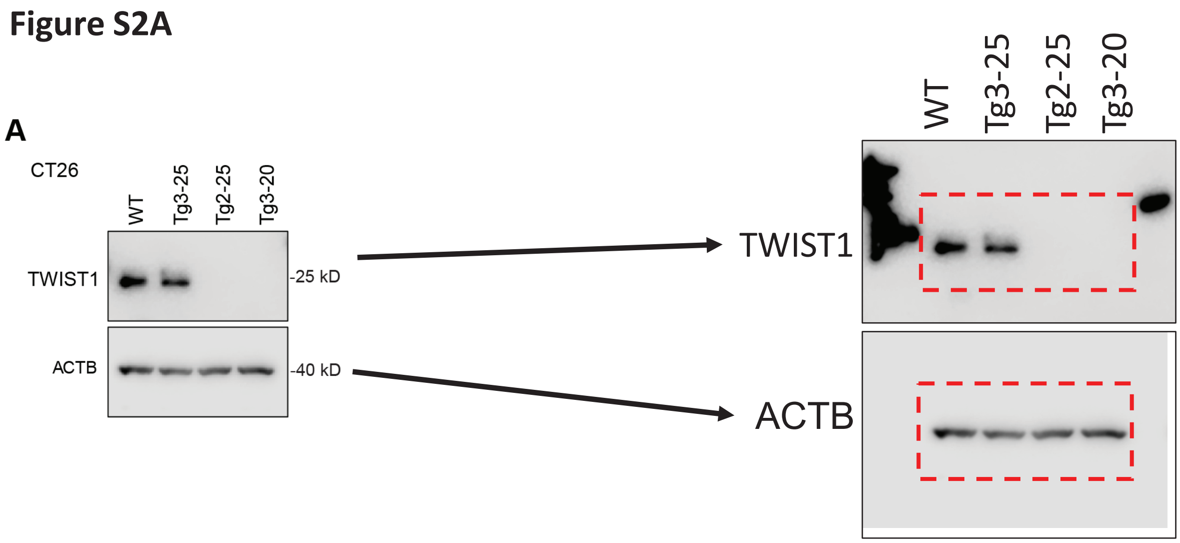

Supplemental Figure 5

Figure S5I

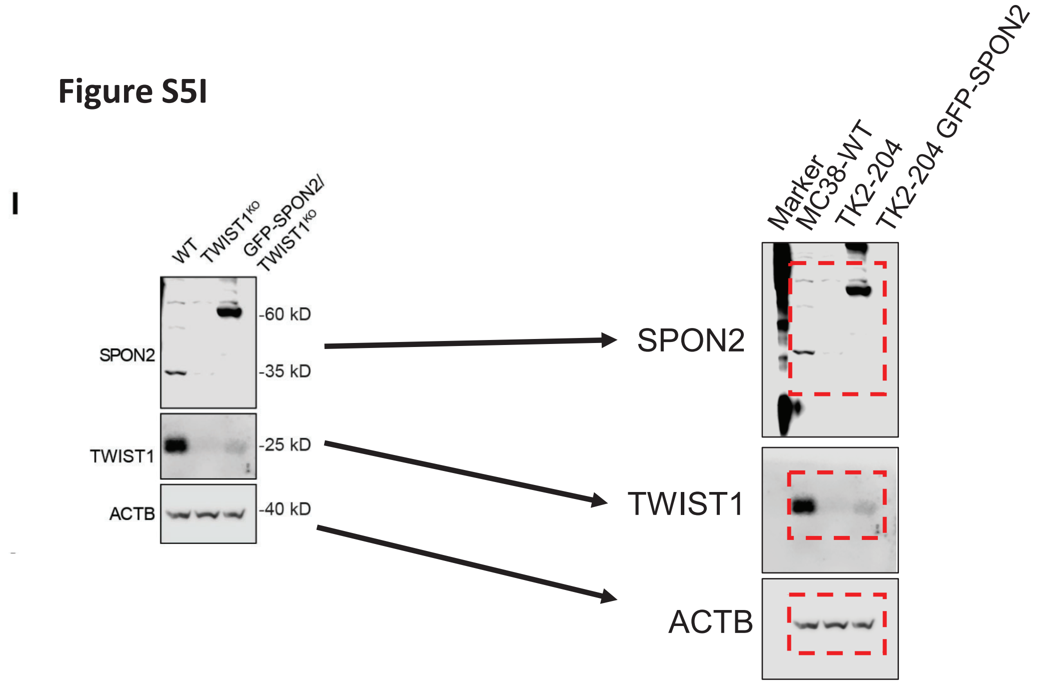

Supplemental Figure 6

Figure S6E

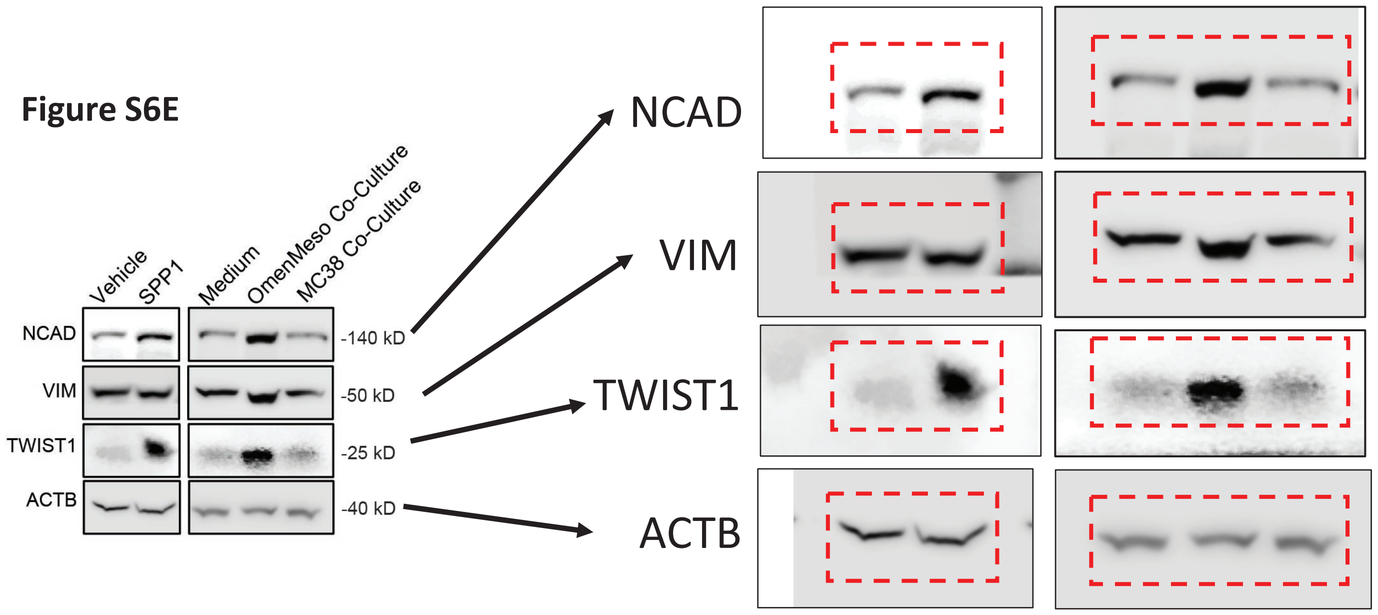

Supplemental Figure 7

Figure S7A

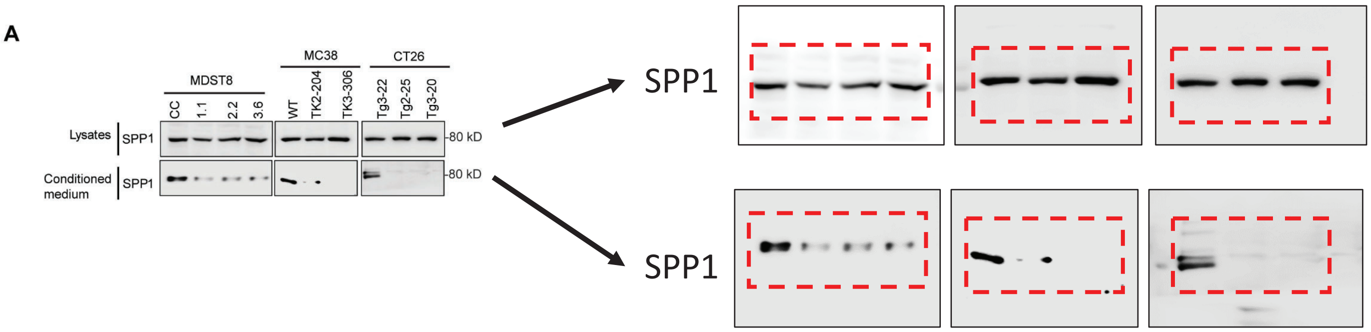

Figure S7D

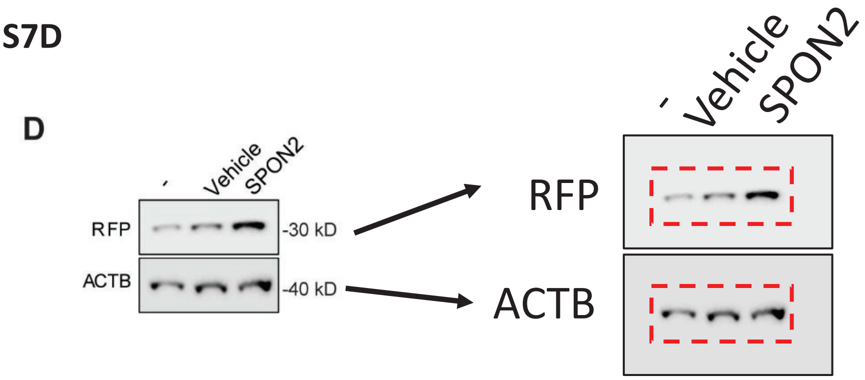

Figure S7D

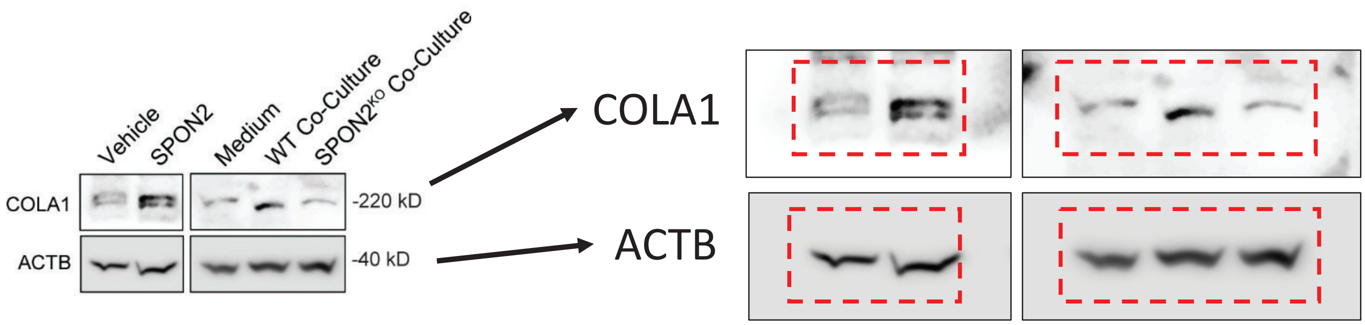

Supplemental Figure 8

Figure S8E

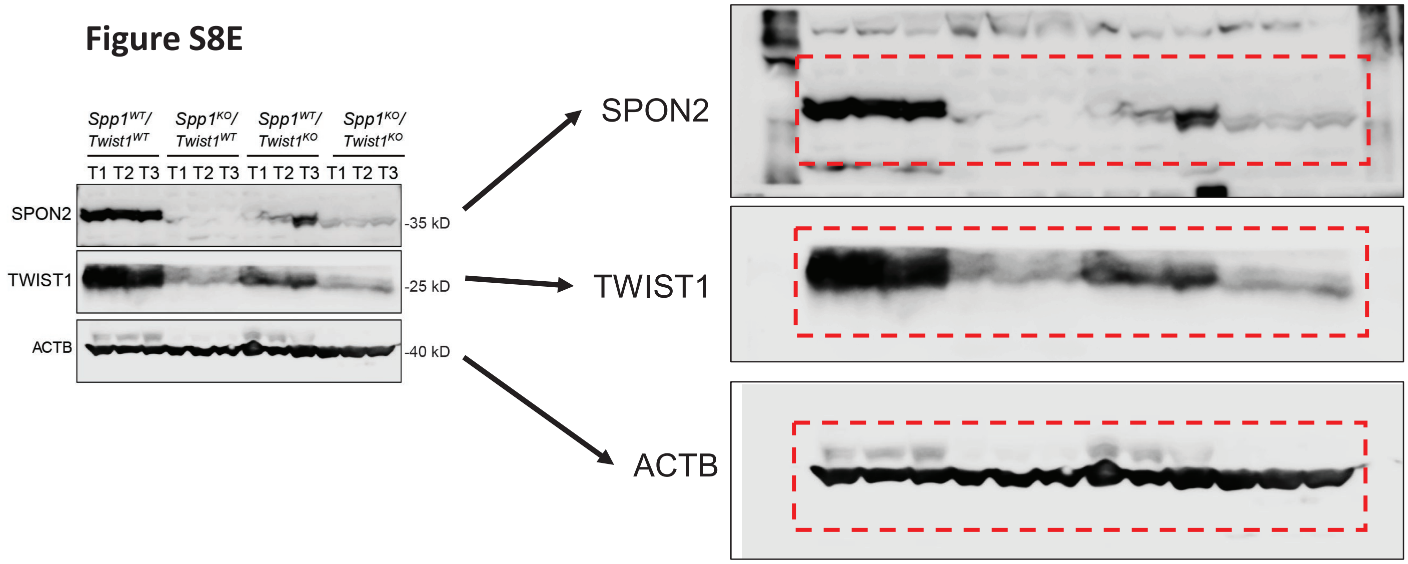

Figure S8G

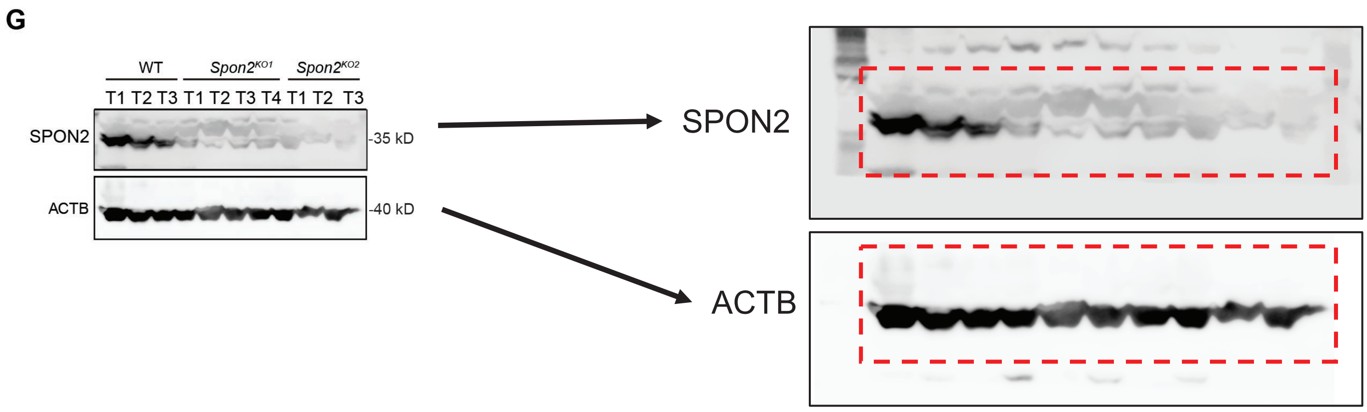

Supplement: Supplementary file 1 — Supplemental Materials [file 41388_2026_3743_MOESM1_ESM.pdf]
